# A comprehensive guide to loop-mediated isothermal amplification, an emerging diagnostic tool for plant pathogenic fungi

**DOI:** 10.3389/fpls.2025.1568657

**Published:** 2025-05-22

**Authors:** Márk Z. Németh, Gábor M. Kovács

**Affiliations:** ^1^ Plant Protection Institute, HUN-REN Centre for Agricultural Research, Budapest, Hungary; ^2^ Department of Plant Anatomy, Institute of Biology, Eötvös Loránd University, Budapest, Hungary

**Keywords:** identification, pathogen detection, DNA-based detection, in-field diagnostics, assay development, SNP detection

## Abstract

The detection and identification of plant pathogenic fungi are crucial for effective plant protection measures. In the past two decades, loop-mediated isothermal amplification (LAMP) has emerged as a simple and cost-efficient tool for plant disease diagnosis, overcoming many drawbacks of traditional and PCR-based methods. LAMP relies on efficient DNA synthesis at a constant temperature, eliminating the need for thermocycling equipment. It is typically more robust, specific, and sensitive than PCR. This literature review summarizes LAMP primer design, reaction protocol development, sensitivity and specificity testing, and result detection methods. We provide examples of how LAMP’s advantages are exploited in disease diagnosis and survey its diverse applications in plant pathogenic fungi research. These applications include the detection, identification, and monitoring of plant pathogenic fungi; the replacement of culture-based methods; the detection of genetic regions associated with functional changes; and the detection of single nucleotide polymorphisms. A comprehensive list of available assays is also provided. Despite its shortcomings—including difficulties with primer design, risks of cross-contamination, and the potential for false positives—LAMP holds significant potential to gain widespread recognition and popularity in the study of plant pathogenic fungi.

## 1 Introduction

Effectively detecting and unambiguously identifying plant pathogenic organisms are fundamental tasks in plant protection and pathology, and they are crucial for developing effective management strategies against plant diseases (McCartney et al., 2003; Ray et al., 2017). Symptoms are traditionally observed for diagnosis (McCartney et al., 2003); however, detection may fail if symptoms do not develop or are too subtle (Khater et al., 2017). Furthermore, many pathogens cause similar symptoms, complicating diagnosis and making it challenging to differentiate between pathogens (Ray et al., 2017). Symptom perception is also subjective (Martinelli et al., 2015), potentially rendering symptom-based detection unreliable. Moreover, symptom-based detection may lead to pathogen identification that is too late for effective management (Le and Vu, 2017).

Both isolation followed by microscopic observation and direct microscopic observation can aid in accurately diagnosing plant pathogens based on their morphological characteristics. While these methods are inexpensive, they are time-consuming and often require experts with specialized knowledge (Ray et al., 2017; Donoso and Valenzuela, 2018).

Alongside traditional approaches, numerous new methods have been developed to detect and identify plant pathogenic organisms (Ray et al., 2017). Among these, nucleic acid amplification techniques are fundamental (Mori and Notomi, 2009), with the polymerase chain reaction (PCR) and its derivatives being the most widely applied (Fakruddin, 2011). Typically, PCR requires a distinct sample preparation step—DNA extraction from infected plant material—before amplification (Dhama et al., 2014). Additionally, PCR-based methods rely on sophisticated thermocycling instruments and require complex post-amplification processing to detect the results (Parida et al., 2008). Due to these factors, at least 3–4 hours are needed for diagnosis (Dhama et al., 2014), but it can take days in some cases (Niessen and Vogel, 2010), and the time to results can be crucial in certain diagnostic situations (Niessen, 2015).

Quantitative real-time PCR addresses this issue by incorporating fluorescent dyes into the reaction, allowing for real-time detection and providing results during or immediately after the reaction (Parida et al., 2008). This method offers clear advantages over conventional PCR (Khan et al., 2018), including greater speed and sensitivity, as well as high-throughput detection (Kralik and Ricchi, 2017). However, it also comes with higher reagent and instrument costs and requires skilled personnel (Panno et al., 2020).

Many user-friendly isothermal DNA amplification methods suitable for diagnostic purposes have been developed over the past few decades (Gill and Ghaemi, 2008). Among these methods, loop-mediated isothermal amplification (LAMP; Notomi et al., 2000) has gained significant interest and has been adapted for a wide range of applications (Fu et al., 2011).

Other isothermal amplification methods exist (Ivanov et al., 2021; Srivastava and Prasad, 2023); however, they are less frequently employed in plant pathology research than LAMP is. LAMP has spread rapidly into pathogen diagnostics due to its simplicity and other appealing advantages (Dhama et al., 2014). Commercially available LAMP-based diagnostic kits have been developed (De Paz et al., 2014), and some LAMP kits are already officially recommended for the routine diagnosis of certain diseases (Mori and Notomi, 2009). LAMP assays can also be integrated into management practices (Zhang et al., 2022a) and hold promise as a tool for in-field diagnosis of plant pathogens (see below).

In this review, we aim to provide an overview of the use of LAMP in the diagnostics of plant pathogenic fungi, including fungal-like oomycetes. We discuss the basic principles of the technique, its advantages, and its limitations. In addition, we provide a checklist of available assays and applications to facilitate the adaptation of LAMP for use in plant protection and pathology.

## 2 Principles of LAMP

The LAMP method relies on special DNA polymerases with strand displacement activity and specifically designed oligonucleotide primers (Notomi et al., 2000). These two components enable highly efficient DNA synthesis at a constant temperature. Strand displacement polymerases make the template strand of DNA available for synthesis by displacing the opposite strand from the double helix (Kamtekar et al., 2004), thereby eliminating the need for denaturation through increased reaction temperature (Nagamine et al., 2001) and, consequently, the need for thermocycling.

In LAMP, at least four primers are used, with two of them required only during the initial stages of the reaction. Commonly called “outer primers,” these two are denoted as F3 and B3 (Notomi et al., 2000). They attach to the 5’ and 3’ adjacent regions of the target on the sense and antisense strands, respectively, similar to PCR primers.

The other two primers, the forward inner primer (FIP) and backward inner primer (BIP; Notomi et al., 2000), have two functionally distinct regions. The 3’ ends of the FIP and BIP are complementary to regions flanked by F3 and B3, while the 5’ ends are identical to two inner regions of the target DNA. These two functionally distinct regions are sometimes joined by a TTTT linker (Notomi et al., 2000), although it is not used in other studies (e.g., Duan et al., 2014c). After the initial steps of LAMP, which require all four primers, only the FIP and BIP are used for DNA synthesis.

**Figure 1:**
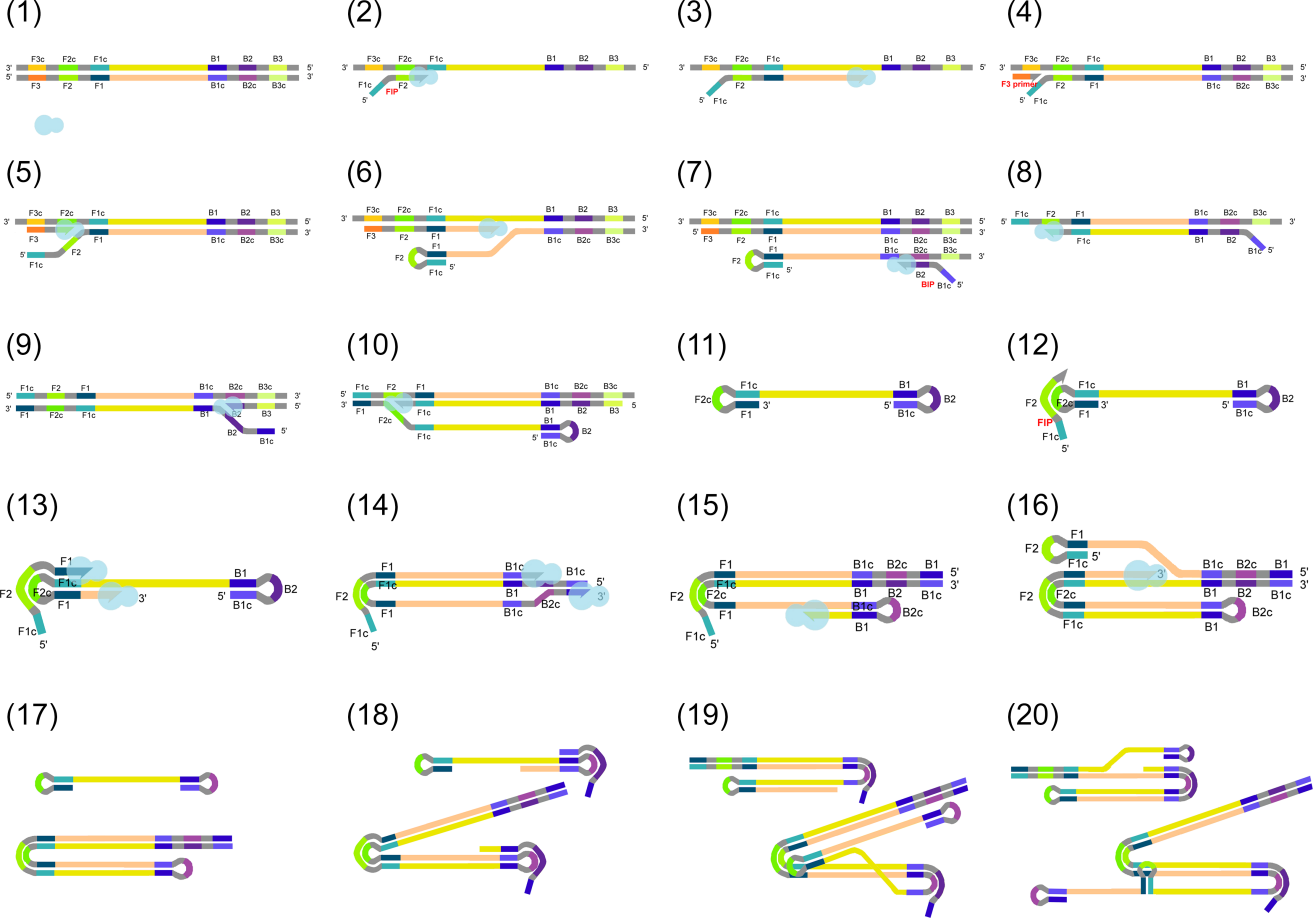
(1) Target DNA and a strand displacement polymerase (shown as a light blue shape) are present in the reaction mix. (2) Firstly, the forward inner primer (FIP) binds to the target DNA with its complementary 3’ end, while DNA polymerase displaces the opposite strand on the original target and polymerization begins and (3) continues in 3’–5’ direction. DNA ends elongated on their 3’ ends are marked as arrows. (4) Then, F3 primer binding and (5) polymerization are also initiated from F3. Due to the activity of the polymerase, the DNA generated in the previous step is displaced. (6) This partially releases the previously synthesized fragment containing FIP. As FIP contains a region complementary to the same DNA strand, the DNA will be self-joined through base pairing to form a loop. (7–10) The same reaction steps also take place on the opposite target strand, involving backward inner primer (BIP) and B3. (11) A DNA fragment with loops on both ends is formed. This structure provides the basis for the upcoming DNA amplification steps. During the amplification, (12) FIP binds to the original target DNA and (13–14) its free 3’ ends, and those of the previously synthesized strands are also elongated. (15–16) Similarly, elongation takes place from the 3’ ends of the later synthesized strands after loop formation. (17–20) In the next steps, LAMP results in the formation of several DNA fragments with different structures, with varying amounts of loops and different DNA lengths. Labels are omitted from steps 17–20 for simplicity.

The steps of the highly efficient DNA synthesis in LAMP were described by Notomi et al. (2000) and are illustrated in **Figure 1**. Due to (i) the strand displacement activity of the polymerase, (ii) the continuous formation of new primer binding sites, (iii) the formation of loop structures, and (iv) the free 3’ ends of the products and primers, DNA synthesis is continuous and highly efficient. Loop structures similar to the initial ones, as well as their complementary forms, are also produced as intermediate products and serve as templates in subsequent steps. Consequently, LAMP produces several DNA fragments with different structures, lengths and varying amounts of loops (Notomi et al., 2000). An animation is available to help explain the principles and steps of LAMP (Notomi et al., 2015; Eiken Chemical Co. Ltd., 2019a).

## 3 Details on primers used in LAMP

The outer primers, F3 and B3, are typically 17–21 nucleotides in length and attach to the 5’ and 3’ adjacent regions of the target intended for amplification. The binding sites (designated as F3c and B3c) are located 0–20 base pairs away from the target region (Dhama et al., 2014). The inner primers, FIP and BIP, contain two functionally distinct regions, usually referred to as F2/B2 and F1c/B1c, which correspond to the 3’ and 5’ regions of these primers, respectively. F2 and B2 are complementary to the F2c and B2c regions of the target DNA and are about 23–24 nucleotides in length. The 5’ ends of FIP and BIP (the F1c and B1c regions) are also about 23–24 nucleotides long and correspond to a region (F2c and B2c) approximately 40–60 nucleotides downstream of the BIP and FIP binding sites (Dhama et al., 2014). The FIP and BIP primers define the target region that will be amplified in large quantities. – Ideally, the length of the target region is between 130–260 base pairs (Notomi et al., 2000).

Correct base pairings are critical for the reaction, so it is crucial to appropriately select primer annealing temperatures (Tm) (Notomi et al., 2000). The Tm of the F2 and B2 regions of FIP and BIP should be around 60–65°C, matching the optimal temperature of the DNA polymerase used (Notomi et al., 2000). The Tm of the F1c and B1c regions should be slightly higher than that of F2 and B2, ensuring the formation of loop structures through base pairing immediately after polymerization (Notomi et al., 2000). The Tm of the outer primers, F3 and B3, should be somewhat lower so that synthesis initiated by the outer primers occurs later than by FIP and BIP (Notomi et al., 2000).

To further support the formation of loop structures—and because the outer primers are indispensable during the initial steps of the reaction (**Figure 1**, steps 4–6 and 9)—the concentration of the outer primers is usually only about one-quarter to one-tenth of the concentration of the inner primers (Notomi et al., 2000). Since these primers are involved only in the initial stages of the reaction, the specificity of the outer primers is less critical than that of FIP and BIP, and minor mismatches in base pairs will not prevent amplification (Niessen, 2015).

One or two additional primers, called loop primers, can also be utilized in LAMP. These primers hybridize to the single-stranded regions on loops, increasing the number of sites where DNA polymerization can be initiated. This increase results in faster LAMP reactions and shorter reaction times (Nagamine et al., 2002).

Swarm primers can further lower the detection limit, improve repeatability, and reduce reaction time, sometimes even in combination with loop primers (Martineau et al., 2017). These primers anneal to the template between the FIP and BIP binding sites on opposite strands and, by swarming the target DNA, improve the accessibility of the binding sites for LAMP primers (Martineau et al., 2017). In the reviewed plant pathology LAMP literature, swarm primers were rarely used. In a case study, swarm primers were shown to enhance the detection of *Fusarium oxysporum* f. sp. *conglutinans*, but this effect was not observed when loop primers were also added to the reaction (Zou et al., 2020).

## 4 Key features and advantages of LAMP

### 4.1 Isothermal reaction

Unlike PCR and related methods, LAMP is conducted at a constant temperature, which is one of its most significant advantages (Dhama et al., 2014). The reaction temperature is typically around 60–65°C, corresponding to the relatively high optimal temperature of the polymerases (Fakruddin, 2011). To maintain the necessary constant temperature, only a simple water bath or heating block is required—an instrument is not required to provide precise and rapid thermal cycling. The need for denaturation through temperature increase is largely alleviated by using polymerases with strand displacement activity (see above; Nagamine et al., 2001).

The most commonly used enzymes are *Bst* polymerase from *Geobacillus* (previously called *Bacillus*) *stearothermophilus* and *Bsm* polymerase from *Bacillus smithi* (Dhama et al., 2014). According to the manufacturers, the optimal reaction temperature is 60–65°C for *Bst* and 60°C for *Bsm*. Some mastermixes also include an enzyme called GspSSD (Jedryczka et al., 2013; Panek and Frac, 2019), which is claimed to provide faster amplification (Le and Vu, 2017). *Bca*BEST DNA polymerase and Z-*Taq* have also been used in LAMP reactions (Notomi et al., 2000). Different polymerases may vary in specificity, sensitivity, thermostability, optimal activity temperature, and inhibitor tolerance, and some also have reverse transcriptase activity (Yang et al., 2024). However, to our knowledge, a systematic comparison of their performance has not been conducted.

### 4.2 Robustness

Since LAMP operates across a wide range of pH levels and incubation temperatures, it is considered more robust than PCR and real-time quantitative PCR (qPCR) methods (Francois et al., 2011). The reaction can proceed even in the presence of inhibitory agents detrimental to PCR, as LAMP typically tolerates these substances (Kaneko et al., 2007). Additionally, LAMP is less likely than PCR to be disrupted by background DNA, meaning DNA from non-target organisms present in the sample (Notomi et al., 2000). Purified DNA extracts are generally not required, as raw DNA extracts produced by crude extraction methods can be used (e.g., Hu et al., 2017).

Setting up the reaction on ice or using freeze racks is usually unnecessary, as LAMP reagents remain active at room temperature and as brief incubation at room temperature does not lead to false positive results (Francois et al., 2011). However, setting up reactions on ice was recommended (Tanner and Evans, 2014) because non-specific polymerase activity may adversely affect reactions under certain conditions (Tanner et al., 2012). Alternatively, unwanted amplification in non-template controls can be avoided by using warm-start polymerases, whose activity is inhibited below 45°C (Poole et al., 2012).

Regarding the storage of reagents, no differences were found in the detection performance of LAMP reactions set up with reagents stored either frozen or at 37°C (Thekisoe et al., 2009). Pre-mixed reaction and primer mixes could be stored in the refrigerator for up to seven days and still yield the same results as fresh reagents (Hu et al., 2023). In a case study simulating delayed initiation of incubation, a 10-minute delay between setting up the reaction mixes and starting incubation did not affect LAMP results (Zou et al., 2020).

### 4.3 Specificity and efficiency

Due to the use of at least four different primers in LAMP, which recognize six distinct target DNA regions, this method is inherently highly specific. The optional loop primers may enhance efficiency, accelerating the reaction. Loop primers may also increase sensitivity (Nagamine et al., 2002), but can sometimes reduce specificity (Duan et al., 2016b, 2018b).

Double-stranded DNA destabilizing agents, such as betaine, L-proline, or the addition of RecA recombinase, can significantly increase reaction efficiency and sometimes also improve specificity (Notomi et al., 2000; Tanner and Evans, 2014; Zou et al., 2020). However, in one instance, omitting betaine increased efficiency (Gao et al., 2016). Using excess dNTPs also enhances efficiency, leading to shorter reaction times (Zou et al., 2020).

The length of the loop in the reaction intermediates affects efficiency, with the ideal loop length being approximately 40 nucleotides (Notomi et al., 2000). The length of the targeted region also influences efficiency. It should be no more than 300 nucleotides (Notomi et al., 2000), although shorter lengths, typically around 130–260 nucleotides, are usually preferred (Dhama et al., 2014). Additionally, the type of polymerase used may impact efficiency, with *Bst* polymerase being superior to another enzyme, Z-*Taq*, in tests (Notomi et al., 2000).

### 4.4 Time- and cost-effectiveness

Consumables for LAMP may not cost significantly less than those for conventional PCR, but the reagents are cheaper (Nguyen et al., 2020). The infrastructural requirements of LAMP are much lower, as sophisticated instruments are unnecessary (Zatti et al., 2019). Reagent and consumable costs for LAMP are estimated to be substantially lower than those for qPCR (Nguyen et al., 2020).

The LAMP reaction is completed in a shorter time than conventional PCR, typically in about one hour (Notomi et al., 2015), which is similar to the duration of a qPCR run. The use of loop primers can reduce the time needed to obtain results to about half an hour (Nagamine et al., 2002), with further reductions possible through optimization. Reaction speed also depends on the type of polymerase used (Niessen, 2015).

After conducting LAMP, it is unnecessary to complete additional post-PCR analysis, such as agarose gel electrophoresis or melting curve analysis. This efficiency, along with the omission of lengthy DNA extraction protocols, allows LAMP to deliver results in a shorter time, thereby increasing the time- and cost-effectiveness of diagnoses (Niessen and Vogel, 2010). Furthermore, LAMP further enhances overall cost-effectiveness by eliminating the need for kits and consumables associated with DNA extraction and gel electrophoresis (Mori and Notomi, 2009).

## 5 Visualization of LAMP reaction results with sequence-independent detection methods

There are numerous methods for visualizing LAMP results (Becherer et al., 2020). These include sequence-independent detection methods, which detect DNA regardless of its sequence, in contrast to sequence-specific detection, which is discussed in the next chapter.

### 5.1 Agarose electrophoresis

Although agarose gel electrophoresis is generally unnecessary for LAMP, it can be used to screen results, especially during assay development. After the LAMP reaction, gel electrophoresis is conducted using relatively concentrated (2%–3%) agarose gels (Notomi et al., 2000; Parida et al., 2008). If the sample is positive, the reaction products of different sizes produce a characteristic ladderlike pattern on the gel (Notomi et al., 2000).

### 5.2 Evaluation by the naked eye

Visual assessment of the turbidity of the reaction mixture after amplification can be performed (Thiessen et al., 2016). During the amplification process, pyrophosphate is produced as a byproduct of DNA polymerization. This pyrophosphate, along with Mg^2+^ ions present in the mixture, precipitates and significantly increases turbidity (Mori et al., 2001). Visual detection of this precipitate is cost-effective and well-suited for field applications (Fukuta et al., 2013).

Alternatively, DNA-binding dyes or colorimetric indicators can be applied (Zhang et al., 2014), either by being added after the reaction (e.g. Tu et al., 2024) or directly to the reaction mixture (Parida et al., 2008).

DNA-binding dyes, which cause a color change in the reaction mixture if DNA amplification occurs, enable direct visualization of results. The ability to detect results with the naked eye without specialized equipment is among the key advantages of LAMP (Dhama et al., 2014). Double-stranded DNA dyes, such as SYBR Green (Chen et al., 2016) and propidium iodide (Hill et al., 2008), can be added after the reaction or, like ethidium bromide, can be added directly to the reaction mixture during setup (Nagamine et al., 2001). Some dyes, such as PicoGreen, must be added after the reaction to avoid reaction inhibition (Tomlinson et al., 2007). A practical method is to place a drop of dye on the inner side of the tube lid, which can then be mixed into the solution by vortexing or centrifuging after the reaction (Lan et al., 2022). Dyes added directly into the tubes before the reaction offer the advantage of reducing the risk of cross-contamination, as the tubes do not need to be opened postreaction (Parida et al., 2008).

Some dyes provide a color change visible in normal light, while others may enhance the color change under UV light (Lu et al., 2015). Additionally, certain indicators, such as malachite green and leuco crystal violet, are initially colorless when the reaction mixture is set up, and they color the mixture only if the samples are positive (Miyamoto et al., 2015).

A decrease in Mg^2+^ concentration in the reaction mixture, corresponding with an increase in DNA concentration and reaction progression (Tomita et al., 2008), can be visualized using metal-ion chelators. One of the most significant developments in LAMP is the use of the hydroxynaphthol blue (HNB) indicator, which initially stains the reaction mixture purple; as the reaction progresses and Mg^2+^ concentration decreases, it changes its color to a characteristic light blue (Goto et al., 2009).

Fluorescent metal indicators like calcein, which indicate a decrease in Mg^2+^ concentration, can also be used (Tomita et al., 2008). However, HNB is considered superior, as it produces a more pronounced color change without requiring fluorescence excitation equipment (Kong et al., 2016). If target DNA amounts are very low and the resulting color change is ambiguous, the results can be confirmed using agarose gel electrophoresis (Yang et al., 2022).

A sensitive detection method involves pH indicators, as the pH in the reaction mixture decreases due to the release of hydrogen ions during dNTP incorporation (Tanner et al., 2015).

A less common, but readily available detection method is based on immunochromatography. This method uses lateral flow devices (LFDs), which simplify result interpretation (Tomlinson et al., 2010b). When running LAMP with antigen-labeled primers, the amplified DNA can be visualized using immunochromatography, with color development on a simple LFD. LFDs have been shown to have a detection limit comparable to agarose gel electrophoresis, which is superior to both SYBR Green-based and turbidity-based detection methods (Patel et al., 2015). LFDs are also optimal for on-site applications (Patel et al., 2015), although they carry a risk of cross-contamination due to the need to open the tubes (Fukuta et al., 2013).

### 5.3 Equipment-based evaluation

While interpreting LAMP results based on color change can be subjective (Patel et al., 2015), instrument-based detection methods can mitigate this subjectivity (Aglietti et al., 2019). One such method involves the photometric detection of turbidity. During LAMP, a precipitate forms, increasing the turbidity of the reaction mix. This change can be measured photometrically to determine whether DNA amplification has occurred (Mori et al., 2001).

Another equipment-based method uses fluorescence detectors sensitive to dyes such as those in commercial mastermixes (Ortega et al., 2018b; Shrestha et al., 2020), SYBR Green I (Peng et al., 2013), and SYTO-9 (Zhang et al., 2013). Changes in fluorescence (Peng et al., 2013) or “time to positive”—namely, the time needed for fluorescence to exceed a certain threshold—are recorded (Ortega et al., 2018b) and used to establish a diagnosis. These data can be applied to create standard curves for absolute quantification (Zhang et al., 2023b; see below).

Another advantage is that fluorescence detection enables the generation of melting curves for the DNA produced during the LAMP reaction. These curves allow direct differentiation of distinct reaction products based on their melting temperatures (Ayukawa et al., 2017). Melting curve analysis can aid in diagnosis even when primers are not entirely specific because it allows the differentiation of amplified products from different organisms (Stehlíková et al., 2020) and the identification of primer dimer byproducts (Siegieda et al., 2021).

## 6 Sequence-specific detection of LAMP results

Sequence-specific detection of LAMP results, primarily using probe-based methods (Zhang et al., 2023a), is becoming increasingly common. This approach measures fluorescence from probes complementary to the target sequence or uses melting curve analysis to distinguish between amplicons of different lengths or sequences.

Sequence-specific detection methods include assimilating probes (Kubota et al., 2011), quenching probes (Ayukawa et al., 2017), and fluorescent loop primers (Komura et al., 2018).

The assimilating probe method relies on fluorescence resonance energy transfer (FRET). A fluorescent probe and a partially overlapping quencher are used (Kubota et al., 2011). During the reaction, the fluorescent probe integrates into the reaction product, causing the quencher to dissociate and the fluorescent signal to increase (Villari et al., 2017).

A universal quenching probe (QProbe) with a joint DNA fragment compatible with the target can be used for specific detection (Ayukawa et al., 2017). The base pairing between the joint DNA and the target, influenced by single nucleotide polymorphisms (SNPs), directly affects quenching, which can be detected by melting curve analysis to reveal the SNPs present in the target (Becherer et al., 2020).

The fluorescent loop primer method uses a fluorescent probe and a quencher (Komura et al., 2018). The latter is designed to hybridize to a position that may contain SNPs. The SNPs present in the sample influence quenching (Komura et al., 2018), which can be monitored based on fluorescence.

## 7 General composition of a LAMP reaction mix

To conduct LAMP, a minimum of 20 μl is recommended as a final reaction volume (Ward and Harper, 2012). Indeed, a final volume of 25 μl is typically used, but 15 µl (e.g., Feng et al., 2015) or 10 μl (e.g., Duan et al., 2016a) may also be used, albeit less frequently. FIP and BIP are used in 0.8 to 1.6 μM concentrations.

While the use of primers purified by high performance liquid chromatography (HPLC) is recommended (Ward and Harper, 2012), using desalted primers has not produced significant differences in some studies (Thiessen et al., 2018; Sedaghatjoo et al., 2021). However, HPLC-purified primers may improve assay rapidity and reproducibility (Tomita et al., 2008). Outer primers (F3 and B3) are typically present at concentrations of 0.2–0.3 μM. To simplify and speed up reaction setup, all primers can be combined in a 10× primer master mix in advance (Karakkat et al., 2018).

Polymerases are used at 4–8 units per 25 μl reaction, dNTPs are used at 400 µM to 1.6 mM concentrations, and betaine is used at 0–1 M concentration. MgCl_2_ is added at 2–8 mM in addition to what is present in the polymerase buffers, but the optimal concentrations of Mg^2+^ and dNTPs are interdependent (Zou et al., 2020).

The final concentrations of indicators are around 8–50 µM for calcein, less than 1 µM for SYBR Green I if used directly in the mix, and 150–300 μM for HNB. 

Typically, 1 μl target DNA is added. The DNA can be a crude extract (see below in Section 9.1.2). If samples contain high levels of contaminants, column-based DNA extraction techniques, DNAzol, phenol-chloroform extraction, or ethanol precipitation should be used (Tanner and Evans, 2014).

For detailed LAMP protocols, readers should consult the paper by Tanner and Evans (2014).

## 8 General method for the development and optimization of LAMP assays

### 8.1 Target loci and primer development

Primers can be designed manually or with software developed for this purpose (Mori and Notomi, 2009). Primers should not have any secondary structure (Parida et al., 2008). Because of this, the manual design of LAMP primers could pose difficulties. Software such as PrimerExplorer has been developed specifically for LAMP primer design purposes to aid the process (Eiken Chemical Co. Ltd., 2019b).

In general, it is recommended that two to four primer sets be tested for the intended target, and the best-performing set in terms of specificity and sensitivity should be chosen (Tanner and Evans, 2014). Efficiency can be assessed by comparing amplification (incubation) length and sensitivity obtained using different primer sets. Specificity is evaluated by the absence of amplification in negative and non-template controls (see below). The selected primer set should yield the fastest and most sensitive positive reactions while minimizing nonspecific amplification (Tanner and Evans, 2014).

Ribosomal DNA genes are the most commonly used target loci for the detection of plant pathogenic fungi with LAMP. About one-third of the reviewed methods target the internal transcribed spacer region of the nucleolar ribosomal DNA (nrDNA ITS) (**Supplementary Table S1**). This distribution is unsurprising, given that this locus is considered the general barcode region for fungi (Schoch et al., 2012). For other genera, such as *Fusarium* and the oomycete genus *Phytophthora*, in which the use of ITS is limited (Geiser et al., 2004; Robideau et al., 2011), other loci like *Ypt1, CYP51C*, and *EF1α* are commonly used, as these provide sufficient variability for differentiating species (Schena et al., 2008; Fernández-Ortuño et al., 2010; O’Donnell et al., 2015).

In several cases, specificity is aided by selecting a locus that does not occur even in closely related species. These loci most commonly include sequence-characterized amplified region (SCAR) markers (Ortega et al., 2018a), specific genes (Malapi-Wight et al., 2016; Tu et al., 2024), random amplified polymorphic DNA sequences (Moradi et al., 2014), or transposons (Katoh et al., 2021). If whole genome sequences are available, genome comparison can aid in identifying unique target regions or genes (Achari et al., 2023; Ouyang et al., 2023). In species lacking sequenced genomes, transcriptome sequencing and analysis may be conducted (Feng et al., 2021).

LAMP may not target only a single species, *forma* sp*ecialis*, race, or strain; by developing primers for amplification of DNA regions present and conserved in a set of target organisms, all those target organisms can be detected with a single primer set. Such primers are available for *Colletotrichum* species (Liu et al., 2021) and for *Clarireedia*, giving a positive diagnosis for three species (Huang et al., 2023).

### 8.2 Reaction protocol development

During the optimization of the reaction mixture components, concentrations of all reagents may need to be finetuned. Since optimization steps leading to the most favorable final reaction setup are not always detailed in publications, the ideal step-by-step optimization protocol remains somewhat elusive. This poses a challenge, as testing all possible reagent concentration combinations may be impractical; however, certain reaction component concentrations are interdependent.

The most prominent example of the latter is that the color of HNB depends on both the Mg^2+^ concentration and the amount of dNTP (Goto et al., 2009). Moreover, Mg^2+^ concentration is also crucial for reaction specificity (Chandra et al., 2016). Generally, the determination of Mg^2+^ concentrations is considered essential (Su et al., 2016). As such, starting with a general reaction mix composition (e.g., taken from an earlier study such as Tanner and Evans (2014)), Mg^2+^ concentrations may first be optimized, with other components remaining constant (Zhou et al., 2021). For this step, Mg^2+^ concentration may be tested in the range of 2–8 (–10) mM, to find the optimal efficiency and yield, balancing between potential non-specific amplification (at higher concentrations) and low or no amplification (at lower concentrations). Optimization is usually done in 1–2 mM increments to find the ideal concentration for robust amplification. When calculating the amount of Mg^2+^ to add to the mix, it is essential to consider that master mixes or buffers typically already contain Mg^2+^ (Yeni et al., 2024).

Optimization of Mg^2+^ concentration may be followed by testing a range of dNTP concentrations (such as (0.2–) 0.5–2 mM in 0.2 or 0.5 mM increments) using the determined Mg^2+^ concentration. Afterward, concentrations of betaine (0–1.6 M), *Bst* DNA polymerase (2–8 U), inner primers (usually 0.8–2 μM), and outer primers (typically 0.2–0.8 μM) can be determined (Zhou et al., 2021). In addition to final primer concentrations, relative concentrations of outer and inner primers may also be optimized (Su et al., 2016). When relative concentrations of primers are tested, the concentration of outer primers is usually fixed and inner primer concentrations are adjusted to different ratios (Winkworth et al., 2020), such as from 1:3 to 1:10. Omitting optimization steps may compromise assay development as suboptimal relative primer concentrations can lead to loss of specificity (Cao et al., 2017).

Orthogonal experiments, which consider possible interactions between reaction factors, can help identify the optimal reaction mixture composition. In this approach, many—but not all—possible reagent combinations are tested, aiding the identification of optimal combinations (Dai et al., 2024).

After the final reaction mix composition is determined, the optimal reaction temperature is usually determined next (typically 60–65°C), after which different incubation lengths (up to 90 mins) are tested (e.g., Ghosh et al., 2015). Necessary incubation length, however, may depend on the amount of polymerase added to the mix: using less polymerase will increase the time needed for the reaction to yield positive results (Gao et al., 2016). The DNA extraction method and the inhibitors present in the samples may also influence the necessary incubation time (Siegieda et al., 2021). However, overly long incubation can lead to false positive results (Le and Vu, 2017).

As an alternative, reaction temperature optimization may be conducted first, followed by reagent concentration optimization (Yeni et al., 2024).

### 8.3 Verification of LAMP reaction products

Confirmation of products may rely on different methods. If the results are tested based on turbidity (Takahashi et al., 2014) or color change in the reaction mix (e.g., Hu et al., 2017), verification by agarose gel electrophoresis is common, at least in the assay development phase. Restriction enzyme digestion may be conducted to check the accordance of resulting fragments with the theoretically expected LAMP products predicted based on the known sequence of the targeted region (Vielba-Fernández et al., 2019). Digested LAMP products may be cloned and sequenced for sequence-based verification (Kong et al., 2016). After extraction from agarose gel, the products can also be sequenced directly using F2 and B2 primers (Sedaghatjoo et al., 2021). Alternatively, LAMP products may be sequenced with F3 and/or B3 primers after amplification by PCR (Duan et al., 2016b).

### 8.4 Primer set specificity testing

Tests of primer set specificity should be conducted with the optimized reaction mix composition and reaction protocol. The specificity of the assay is often checked with DNA originating from other plant pathogens occurring in a similar niche or on the same host (Shen et al., 2016; Manjunatha et al., 2018; Zhang et al., 2024) or with the DNA of closely related species (Kong et al., 2016; Sunani et al., 2019). In an ideal case, specificity is also checked against the DNA of the host plant (Niessen and Vogel, 2010; Niessen et al., 2012). One reliable method for testing a wide range of species for cross-reactivity uses DNA originating from environmental samples, containing DNA from hundreds of potentially cross-reacting species (Malapi-Wight et al., 2016). Occasionally, however, entirely specific amplification cannot be obtained (Niessen et al., 2012), and therefore, several sets of primers, potentially targeting other loci, may need to be tested (Shen et al., 2017; Madihah et al., 2018).

Using dimethyl sulfoxide (DMSO) as an additive or employing a touchdown LAMP protocol (or both) may increase both specificity and sensitivity (Wang et al., 2015).

Real-time detection can allow one to identify specific products with melting curve analysis (Tomlinson et al., 2013), potentially rendering less specific primers suitable for diagnosis. Alternatively, real-time detection can exclude aspecific amplification by setting a cut-off for the amplification reaction length (Vielba-Fernández et al., 2023). This approach is similar to the cycle cut-off in real-time PCR (Cai et al., 2008). Moreover, the developed LAMP primers may remain suitable for detection and disease diagnosis even if only partially specific. Examples include cases where they do not amplify DNA from other pathogens on the same host (Yang et al., 2020) or amplify off-target fungi that occur only in extremely low ratios (Xu et al., 2021).

### 8.5 Sensitivity assays

Reaction sensitivity is usually measured by running serial diluted target DNA to determine the smallest amount or concentration of DNA detected by LAMP (Zhang et al., 2019). The DNA may originate from a pure culture of the pathogen (e.g., Xiong et al., 2021) or from infected plant tissues, thus containing the DNA of both pathogens and host plants (Chandra et al., 2015). Alternatively, a target fragment cloned in a vector may be used for serial dilution and sensitivity testing (Pu et al., 2014). Another approach involves using serially diluted spore suspensions to assess sensitivity (Hong-min et al., 2023).

A similar sensitivity test is often done with a PCR method as a control. LAMP sensitivity compared to PCR is usually expressed as how many times more diluted DNA is amplified by LAMP, which PCR cannot amplify. Notably, however, the PCR used for such comparisons is usually not (highly) optimized (Niessen et al., 2012) or is not the most commonly applied method for diagnosis of the given pathogens, but rather a protocol used only for this type of sensitivity comparison assay. In such tests, outer primers designed for LAMP may be used as primers for the PCR (e.g., Liu et al., 2019). Another approach for sensitivity comparisons is to analyze the same set of samples with LAMP and with other methods, such as PCR (Xiong et al., 2021); real-time quantitative PCR (Zhang et al., 2022b); and alternative, non–DNA-based methods, such as baiting (Tong et al., 2021) or direct isolation (Ren et al., 2021). The results obtained by different methods are then compared (Lan et al., 2020).

Comparisons indicate that the sensitivity of LAMP for the detection of a given pathogen is at least the same as PCR (Notomi et al., 2000; Gao et al., 2016) and lies in the range of 100 fg–50 ng/µl, but in about 80% of the examples LAMP is more sensitive by several orders of magnitude (e.g., Ghosh et al., 2015) and can detect DNA amounts as low as 0.1–1 fg (**Supplementary Table S1**). Real-time–based fluorescence detection tends to offer slightly better sensitivity (**Supplementary Table S1**), but the difference is not striking. More than two-thirds of assays using real-time fluorescence detection have their detection limit in the femtogram or picogram magnitude. In turn, two-thirds of SYBR Green and HNB assays (evaluated with the naked eye) detect the DNA amounts in the picogram magnitude (**Supplementary Table S1**).

## 9 Application of LAMP in plant pathology

The main driving forces in developing LAMP methods are simplifying and accelerating the detection of plant pathogens and improving cost efficiency (Tomlinson and Boonham, 2008). Methods are also developed for identification purposes (e.g., Niessen and Vogel, 2010). The first uses of LAMP in plant pathology include the detection of plant pathogenic viruses and bacteria. For an introduction to these works on the detection of bacteria, viruses, and plant pathogenic nematodes, consult other reviews (Tomlinson and Boonham, 2008; Panno et al., 2020; Ahuja and Somvanshi, 2021; Babilônia et al., 2024).

In the following section, we summarize a comprehensive list of LAMP methods for identifying plant pathogenic fungi and oomycetes, and other applications of the technology, in light of its main advantages and applications (**Supplementary Table S1**). Several of the surveyed assays (about 20%) target *Fusarium* species and oomycetes (**Supplementary Table S1**).

### 9.1 Characteristics of LAMP exploited in plant pathology

One of the general advantages of LAMP is shorter reaction lengths (Zeng et al., 2017), and this advantage holds for most if not all of the cases reviewed here. The sensitivity of the published assays is at least the same as that of PCR-based diagnostics, but in most cases, it is better (**Supplementary Table S1**).

#### 9.1.1 Ease of use, cost-effectiveness, and low instrument needs

Owing to the isothermal reaction conditions and the immediate results provided, LAMP assays are characterized by their ease of use, cost-effectiveness, and low instrument needs. One of the first uses of LAMP for detecting a plant pathogenic Oomycete was an assay targeted at *Phytophthora ramorum*, the causal agent of sudden oak death (Tomlinson et al., 2007). Despite LAMP having slightly less sensitivity than a real-time PCR, it was praised for its basic equipment requirements and ease of endpoint detection with the naked eye (Tomlinson et al., 2007). Researchers have indicated its potential to be applied by nonspecialized staff or in laboratories with limited equipment or facilities (Huang et al., 2023) and resources, as well as its suitability for use in the field (Tomlinson et al., 2007). The LAMP method developed for the detection of *Ph. melonis*, a pathogen species causing blight, dieback, or rots of diverse host species of Cucurbitaceae, was highlighted for its simplicity and low costs (Chen et al., 2013). A LAMP assay developed for the detection of *Didymella bryoniae*, a pathogen causing gummy stem blight to cucurbitaceous plants, was also recommended as an easy-to-perform diagnosis even for amateur users or laboratories without elaborate equipment (Yao et al., 2016).

#### 9.1.2 Compatibility with simple DNA extraction methods or material placed directly in the reaction mixture

As LAMP methods are generally more robust than PCR–based methods, they can often be used after a simplified DNA extraction resulting in a crude DNA extract, or even without DNA extraction. An early use of LAMP was detecting *F. graminearum*, the causal agent of fusarium wilt and producer of numerous mycotoxins (Niessen and Vogel, 2010). By placing a small fragment of the colony or infected barley seeds directly into the mix, the assay can be used to test whether the fungal isolate belongs to the *F. graminearum* species without DNA extraction (Niessen and Vogel, 2010).

A LAMP-based method to detect *F. oxysporum* f. sp. *lycopersici* (the forma specialis of *F. oxysporum* infecting tomato) is also possible without prior DNA extraction (Almasi et al., 2013). A LAMP method targeting *Ph. sojae* was published, in which rapid lysis-based crude DNA extracts, including DNA from soil samples, were used (Zhao et al., 2015). Similarly, a sensitive LAMP method has been developed as a rapid and cost-effective method to monitor *Ph. capsici*, which causes blight and fruit rot in peppers and other Solanaceous and Cucurbitaceous hosts. This assay could detect the pathogen from infected plants following a relatively crude DNA preparation method (Dong et al., 2015). Similarly, a LAMP assay could detect *Guignardia citricarpa*, associated with citrus black spot, from crude DNA extracts (Tomlinson et al., 2013).

For the detection of *Pythium helicoides*, a LAMP method was shown to provide optimal results from infected plant material cut into segments, whole infected seeds, or fungal colonies without DNA extraction by simply vortexing material and using the supernatant as the target (Miyake et al., 2016). The same DNA extraction method was used with the LAMP assay developed for *Py. aphanidermatum* (Fukuta et al., 2013). Seeds were used as a starting material to produce crude DNA extracts for the detection of *F. oxysporum* f. sp. *lactucae* (Ortega et al., 2018a). A LAMP assay using samples prepared in 5–10 minutes significantly accelerated the fungicide resistance risk assessment of *Botrytis cinerea* (Hu et al., 2017).

Nitrocellulose membranes of commercial LFD may also be used for rapid DNA extraction, after which the membranes can be used for amplification (Tomlinson et al., 2010b; Wu et al., 2019). Recently, the detection of tea anthracnose pathogen by LAMP through rapid, filter paper-based DNA extraction was published (Zou et al., 2024). When performed following a rapid DNA extraction, LAMP can significantly accelerate the diagnostic process.

### 9.2 Uses of LAMP in plant pathology

#### 9.2.1 Pathogen detection

In the initial phases of several diseases, characteristic symptoms may be lacking, even if the pathogen has already been established (Shen et al., 2016). In such situations, LAMP may aid diagnosis. For example, assays are available to detect rust species in the initial phase after infection, as early as one to two days post-infection. These include assays targeting *Puccinia striiformis* f. sp. *tritici* (causal agent of wheat yellow rust (Huang et al., 2011; Aggarwal et al., 2017), *P. triticina* (the pathogen causing wheat leaf rust (Manjunatha et al., 2018); and *Sporisorium scitamineum* (causal agent of rust on sugarcane (Shen et al., 2016; Su et al., 2016). An assay detecting *F*. *oxysporum* f. sp. *cubense* race 4 can be used on banana plants that are not yet showing wilting symptoms (Li et al., 2013). Similarly, LAMP primers targeting *Ph. infestans* detect the pathogen from symptomless plants infected only one hour before testing, outperforming alternative methods (Khan et al., 2017). The presence of *C. gloeosporioides* on symptomless guava fruits can be verified with a LAMP assay, giving results similar to the isolation of the pathogen (Lan et al., 2020).

Sequence-specific detection methods (the assimilating probe method; see above) were developed for the detection of the *M. oryzae* pathotype Triticum (Yasuhara-Bell et al., 2018), *Ph. infestans* (Si Ammour et al., 2017), and *Fusarium circinatum* (Stehlíková et al., 2020) from their hosts.

LAMP can identify pathogens in the broader environment, not just in association with the host plant. This identification includes pathogens present in their respective vectors. For example, *Raffaelea lauricola*, a causal agent of wilt disease of lauraceous hosts, is spread by a beetle (*Xyleborus glabratus*). To detect the pathogen directly from the vector (and also from hosts), a LAMP method was developed (Hamilton et al., 2020). Additionally, weeds growing on and around rice paddies and potentially carrying *Sarocladium oryzae*, the causal agent of rice sheet rot, were assayed with the method originally developed to detect the pathogen from rice (Logeshwari et al., 2022; Choudhary et al., 2022). *Rhizoctonia solani* could be detected not only from rice plants, but also from the soil with a LAMP method (Choudhary et al., 2020). Similarly, a LAMP method could detect *Phytopythium vexans* zoospores from water (Ghimire et al., 2023).

#### 9.2.2 Identification of pathogens causing aspecific symptoms

Even when symptoms are present, identifying the pathogen can be challenging due to different pathogens causing similar symptoms. One such example is the similarity between the general symptoms of fusarium wilt in chickpeas and those of dry root rot (Ghosh et al., 2015). This was the reason for developing a LAMP method to identify *F. oxysporum* f. sp. *ciceris*, the causal agent of chickpea fusarium wilt (Ghosh et al., 2015). For detection of *Py.* sp*inosum*, a species causing seedling and root rot, which are quite similar to those caused by other pathogens, a LAMP method is available (Feng et al., 2019). Rust fungi infecting sugarcane (*P. kuehni* and *P. melanocephala*) are hard to differentiate in the initial phases of the disease because of the similar symptoms caused. A LAMP assay targeting *P. kuehnei* supports quick pathogen identification, which would take significantly longer using microscopic investigation (Chandra et al., 2016). Although *Magnaporthe oryzae* pathotype Triticum causes symptoms similar to *F. graminearum*, a LAMP method (Yasuhara-Bell et al., 2018) can differentiate between these pathogens.

#### 9.2.3 Identification of morphologically similar fungi

Diagnostic analysis of morphologically similar species is complicated, and the commonly used sequence-based identification is time-consuming and expensive. In such cases, LAMP can aid identification. To detect *F. fujikuroi*, the causal agent of bakane disease in rice, at least three different LAMP methods were developed (Rong et al., 2018; Sunani et al., 2019; Zhang et al., 2019). Aside from this species, the disease may also be caused by others, such as *F. proliferatum*, whose identification through morphological analysis is both time-consuming and labor-intensive (Zhang et al., 2019). As an alternative, specific LAMP assays are available (Rong et al., 2018; Zhang et al., 2019) to detect *F. proliferatum* and *F. fujikuroi*.

#### 9.2.4 Inoculum monitoring and disease forecast

LAMP can be used for both inoculum monitoring and supporting disease prevention efforts. The precise timing for plant protection measures is necessary for effective disease management. The LAMP assay developed for the detection of *F. oxysporum* infecting *Dendrobium officinale* was shown to support plant protection measures, as after positive diagnosis of asymptomatic plants, fungicides can be deployed immediately (Xiao and Li, 2021).

A LAMP method was designed to detect airborne spores of *M. oryzae* causing gray leaf spot disease on ryegrass (Villari et al., 2017). Spores collected in spore traps in field plots could be detected with the method 12 days before symptoms appeared on ryegrass. Similarly, a method for detecting spores of *Uromyces betae*, the causal agent of sugar beet rust, was used to monitor inocula collected on air spore tapes (Kaczmarek et al., 2019). A LAMP method for surveillance of the *M. oryzae* Triticum pathotype has also been developed to detect and prevent potential outbreaks (Yasuhara-Bell et al., 2018). Surveillance needs have also led to the development of a LAMP assay targeting *Pyrenopeziza brassicae*, the causal agent of light leaf spot in *Brassica* species (King et al., 2018). LAMP assays are also available to detect and monitor the quarantine pathogens *Ceratocystis platani* and *Ph. ramorum*, the causal agents of canker stain disease in plane trees and sudden oak death, respectively (Aglietti et al., 2019). For the specific detection of the airborne inoculum of the *Lolium perenne* pathotype of *M. oryzae* (causing grey leaf spot on ryegrass), a sequence-specific detection with an assimilating probe (see above) was used (Villari et al., 2017).

Inocula of certain pathogenic fungi may be present in the soil. LAMP methods were developed to detect those from the soil, for example for *F. oxysporum* f. sp. *fragariae* infecting strawberry (Katoh et al., 2021); *Peronophythora litchii* infecting lychee (Kong et al., 2021); and *Ph. cinnamomi*, which infects a wide range of hosts (Tong et al., 2021).

#### 9.2.5 Supporting or replacing culture-based methods

LAMP can support or substitute conventional culture-based methods. For example, a LAMP assay to detect *G. citricarpa* helps overcome difficulties with traditional and culture-based methods, which consume more time and lack reliability due to false negatives, as *G. citricarpa* is easily overgrown by other fungi (Tomlinson et al., 2013).

Certain studies have compared culture-based and LAMP-based methods, such as a LAMP assay for detecting *Ph. sojae*, the causal agent of soybean root rot. LAMP showed improved sensitivity over traditional culture-based and PCR-based methods (Dai et al., 2012). A LAMP method targeting *C. gloeosporioides* was also more sensitive than isolation (Lan et al., 2020). A LAMP assay detected *Didymella bryoniae*, the causal agent of gummy stem blight, in infected cucurbit seed batches (Tian et al., 2017). The same seed batches were also tested using the standard blotter assay, which involves moist chamber incubation, seedling observation, and microscopic examination of fungal structures. The results matched those of LAMP (Tian et al., 2017). As the blotter method requires large seed samples and takes several days, LAMP may offer advantages in speeding the process. Similar comparisons were conducted with LAMP targeting *F. fujikuroi* and *M. oryzae* in rice seed, showing the reliability of the developed LAMP methods in surveillance (Ortega et al., 2018b).

Three diagnostic methods, namely isolation from infected tissues as well as PCR- and LAMP-based detection of two necrotrophic pathogens, *B. cinerea* and *S. sclerotiorum*, were compared (Duan et al., 2014a, b). For the detection of *S. sclerotiorum*, the results of the three methods agreed (Duan et al., 2014a). The ratio of positive samples was higher with LAMP and the traditional culture-based method than with PCR in *B. cinerea* diagnostics. Thus, the application of LAMP significantly improved detection efficiency, and is therefore recommended for rapid and early diagnosis of these pathogens (Duan et al., 2014a, b).

Complete agreement was observed when the results of the conventional method (plate testing) were compared with those of the developed LAMP assay targeting an SNP associated with fungicide resistance in *B. cinerea*. Moreover, the LAMP assay could be performed in a shorter time (Hu et al., 2017). The same efficiency was demonstrated in tests targeting SNPs in *B. cinerea* associated with a second group of fungicides (Duan et al., 2018a; Liu et al., 2019), producing results comparable to plate-based tests.

#### 9.2.6 Functional studies

LAMP may also be used to detect the presence of a particular DNA fragment related to certain phenotypes and functions in the organism in question. For example, mating-type idiomorphs—determinants for fungal sexual reproduction—can be screened with LAMP. Such a method was developed to identify mating types of *Oculimacula acuformis* and *O. yallundae* (King et al., 2021).

In *Monilinia fructicola*, resistance to demethylase inhibitor-type fungicides (DMIs) can be caused by overexpression of a demethylase gene (*MfCYP51*). If an inserted fragment (called Mona) is present in the upstream flanking region of the gene, the gene will be overexpressed, rendering the fungus resistant to DMIs. Although many different copies of Mona may be present in the genome, precise LAMP primer design enabled the specific detection of strains carrying the Mona element upstream of *MfCYP51* (Chen et al., 2019).

Strains of *B. cinerea* that carry an intron in the *cytochrome b* gene are associated with a lower risk of developing resistance to quinone outside inhibitor fungicides. An assay targeting this intron (Hu et al., 2017) aims to support resistance risk assessment.

The same principle was used to detect the toxin-producing *Alternaria alternata* tangerine pathotype by targeting its toxin-production gene with a LAMP assay (Moghimi et al., 2016), as well as the ochratoxin A-producing *Aspergillus carbonarius* stains from grapevine (Storari et al., 2013).

With LAMPs targeting race-specific fragments, pathogen races can be identified. *Fusarium oxysporum* f. sp. *cubense* race 4, which attacks banana plants, can be specifically detected via such an assay (Li et al., 2013). A slightly different approach is followed to detect race 1 of *F. oxysporum* f. sp. *lycopersici*. Three LAMP primer sets were developed targeting three different genes, present together only in this race; thus, the race is identified if all three primer sets give positive results (Ayukawa et al., 2016).

#### 9.2.7 Quantitative applications

Turbidity or fluorescence changes in the LAMP mix can be monitored to track reaction progress, with fluorescence detected using intercalating dyes or probes (Mori et al., 2004; Tomlinson et al., 2007). Using real-time logging and reference samples with known DNA concentrations or DNA copy numbers, LAMP can also enable quantification (Mori et al., 2004; Peng et al., 2013).

The time needed to reach a certain threshold of turbidity or level of fluorescence correlates linearly with the logarithm of the DNA amount added to the reaction (Mori et al., 2004; Tomlinson et al., 2007). LAMP methods leveraging this principle are referred to as quantitative real-time LAMP (qLAMP; Villari et al., 2017).

Examples of the uses of qLAMP are the quantification of spores of *Uromyces betae* (Kaczmarek et al., 2019) and those of the grapevine powdery mildew fungus *Erysiphe necator* (Thiessen et al., 2018). The DNA amount of *F. oxysporum* s. sp. *niveum* (Peng et al., 2013) and *P. inflatum* present in soil samples (Cao et al., 2016) were also measured by the respective qLAMP methods. Quantification of *U. maydis* present on different maize lines is also available (Cao et al., 2017). Additionally, qLAMP methods are potentially available for quantification of *Ph. ramorum* (Tomlinson et al., 2007), *B. cinerea* (Tomlinson et al., 2010a), *F. oxysporum* f. sp. *cubense* Tropical Race 4 (Zhang et al., 2013; Peng et al., 2014), *Ph. infestans* (Si Ammour et al., 2017), *F. circinatum* (Stehlíková et al., 2020), and *Ustilaginoidea virens* (Zhang et al., 2023b). However, in most cases, the authors of these works did not conduct quantification, per se, but they nevertheless demonstrated its possibility, as the correlation between the target DNA amounts and threshold times was proven.

Similar to the fact that conventional LAMP is less sensitive to reaction inhibitors, real-time LAMP is less sensitive to inhibitors than is quantitative PCR (Tomlinson et al., 2010a; Peng et al., 2013). In a comparison of real-time and conventional LAMP methods, their sensitivity was equal (Rizzo et al., 2022).

#### 9.2.8 SNP detection

LAMP’s superior specificity supports its deployment in detecting SNPs (Parida et al., 2008). In such assays, the polymerase continuously verifies the presence or absence of the targeted SNP (Fakruddin, 2011) and amplifies only the desired variant.

Originally, SNP detection via LAMP was achieved with both FIP and BIP primers hybridizing to the targeted SNP (Iwasaki et al., 2003; Eiken Chemical Co. Ltd., 2019c). If both primers carry a nucleotide correctly pairing only with the SNP in their 5’ end (F1c and B1c, respectively), no amplification occurs if the target nucleotide is absent. The dumbbell structure of LAMP forms, but further polymerization is attenuated or delayed because the dumbbells have only mismatched step-loop structures, and thus, discrimination of SNPs is achieved (Iwasaki et al., 2003).

Interestingly, to our knowledge, this principle has not been used for SNP detection in plant pathogenic fungi because only a single BIP or FIP primer (instead of both) hybridizing to the variable position has been proven to assure necessary discrimination between wild-type and mutant alleles. Oftentimes, an approach similar to allele-specific PCR was followed. Only the 5’ end nucleotide of the BIP or the 3’ end nucleotide of the FIP corresponds to the SNP to be detected in those works. In this method, LAMP dumbbell structures do not form (or do so only rarely), as there are no decent priming sites for starting highly efficient displacement amplification. Artificially introduced mismatches in the primers are often necessary to provide selectivity (see below).

In phytopathology, LAMP differentiating SNPs (SNP-LAMP) can detect pathogen races differing only in SNPs (Ayukawa et al., 2017; see above). However, SNP-LAMP is most widely used to detect SNPs leading to fungicide resistance (**Supplementary Table S2**). Such a method for differentiating carbendazim-resistant and -sensitive strains of *F. graminearum* was developed (Duan et al., 2014c). The method targeted the most common SNP of *F. graminearum* associated with carbendazim resistance, the point mutation of the second nucleotide in codon no. 167 of beta tubulin (F167Y). The strategy relied on developing a complete set of LAMP primers (F3, FIP, BIP, and B3), with the 5’ end of BIP corresponding to the mutated nucleotide associated with fungicide resistance. In addition, to avoid amplification of the wild type, artificially mismatched nucleotide positions were introduced in the different BIP primers to be used with the same primer set. Altogether, seven different BIP primers were tested, and only one was found to provide the necessary specificity, distinguishing wild-type and mutated alleles (Duan et al., 2014c). This BIP primer had two nucleotide mismatches compared to the exact matching BIP primer developed originally. The new screening method was verified with traditional *in vitro* growth tests on a fungicide-containing medium and sequencing. It was then applied to demonstrate its ability for large-scale resistance monitoring (Duan et al., 2014c).

The same strategy of the primer design was applied to detect a point mutation in beta-tubulin at codon 198 (E198A) of *Sclerotinia sclerotiorum*, responsible for carbendazim resistance in this fungus (Duan et al., 2015). In that work, eight FIP primers were tested for specificity, and four of them, all containing one or two artificially introduced mismatches, provided the required specificity. After extensive testing and application of the method, it was highlighted that it is simpler and faster than other alternatives to detect E198A of *S. sclerotiorum*, as detection can be achieved in only one hour (Duan et al., 2015). Other studies, targeting F200Y mutations of *F. asiaticum* and *S. sclerotiorum*, and E198A in *Podosphaera xanthii*, markers of carbendazim resistance in these fungi, also followed the strategy of introducing artificial mismatches into the 3’ end of FIP primers (region F2) (Duan et al., 2016a, b; Vielba-Fernández et al., 2019). A method for detecting the F200Y mutation of the beta-tubulin gene of *B. cinerea* was also developed and found to be 100 times more sensitive than conventional PCR (Duan et al., 2018a). To detect SNPs in the *sdhC* gene of *Po. xanthii*, conferring resistance to succinate dehydrogenase inhibitor (SDHI) fungicides, two different primer sets were developed (Vielba-Fernández et al., 2021). An assay is also available to detect the most common marker of SDHI resistance in *B. cinerea* (Fan et al., 2018).

LAMP methods targeting a single mutation are suboptimal if the same site may have more than two common alleles (e.g., wild type and more than one mutated version). Optimal primer planning, however, makes it possible to design LAMP assays targeting more than one SNP at once (Duan et al., 2018b). Most carbendazim-resistant *B. cinerea* strains have a mutation at the codon no. 198 of beta-tubulin, but many other SNPs that lead to high resistance levels were also described. Introducing a degenerate nucleotide position in the FIP, a single assay could simultaneously detect three different point mutations (E198A, E198K, and E198V) (Duan et al., 2018b). Alternatively, three different FIPs, each developed for a specific mutation, are available (Fan et al., 2019).

In addition to mismatch primers, the application of probes coupled with melting curve analysis also provides a powerful method for SNP detection. This approach allowed specific detection of *F. oxysporum* f. sp. *lycopersici* race 3 based on unique SNPs present in this race only (Ayukawa et al., 2017). To detect SNPs associated with benzimidazole resistance of *Fusarium* spp. strains causing head blight, the fluorescent loop primer method (see above) was used (Komura et al., 2018). This method enables the differentiation of three β2-tubulin SNPs, each conferring some resistance to benzimidazole fungicides (Komura et al., 2018).

#### 9.2.9 Point-of-care or in-field diagnosis of plant pathogens

Point-of-care diagnosis requires an easy, user-friendly, and lab-independent DNA extraction method with minimal or no equipment requirements and straightforward scoring of results (Ivanov et al., 2021). Because of its general characteristics detailed above, LAMP is regarded as a promising method for in-field diagnosis (Donoso and Valenzuela, 2018; Gomez-Gutierrez and Goodwin, 2022).

Since LAMP is highly robust, the tedious lysis, extraction, and purification steps typically required in sample preparation can be omitted (Moehling et al., 2021). The use of fast DNA extraction reagents (Hu et al., 2017), crude DNA extraction (Ortega et al., 2018a), or simply vortexing (Miyake et al., 2016) may be sufficient. Interestingly, LFDs can also be used for fast and efficient DNA preparation (Tomlinson et al., 2010b).

Concerning running reactions lab-independently, portable devices (Myrholm et al., 2021; Tonka et al., 2022) make point-of-care diagnosis possible (Thiessen et al., 2018). Reagent transportation and storage are also key aspects of in-field diagnosis. According to some reports, LAMP reagents remain active at room temperature (Francois et al., 2011), and no difference was detected in LAMP performance between reactions set up with reagents stored frozen versus those in unrefrigerated storage (Thekisoe et al., 2009). However, stabilizing reagents for storage may be desirable (Donoso and Valenzuela, 2018). Prolonging shelf-life is possible, for example, by drying and using trehalose as a cryoprotectant (Hayashida et al., 2015), but this approach remains uncommon in plant pathology.

LAMP’s ability to offer easy, fast, and equipment-free visualization of products renders it suitable for in-field scoring. Most commonly, HNB and SYBR Green I scoring by the naked eye is used for these methods. LFDs are also optimal for on-site applications (Tomlinson et al., 2010b; Patel et al., 2015).

The “microdevice” developed for the detection of *M. oryzae* and *Sarocladium oryzae* (Prasannakumar et al., 2021) represents the first example of a LAMP-based microdevice to detect plant pathogens, and it is potentially deployable in field conditions.

## 10 Limitations of LAMP

### 10.1 Difficulties with primer design

Because of the two functional segments of FIP and BIP, primer design for LAMP is more complex than that of PCR primers (Tomlinson and Boonham, 2008). Primer design is further complicated by the need to select a target region with sufficient specificity for the intended application and an optimal length—long enough to include primer recognition sites but not so long as to hinder efficient LAMP amplification (Notomi et al., 2000; Wong et al., 2018). These difficulties can be overcome with computer programs such as PrimerExplorer (Eiken Chemical Co. Ltd., 2019b), which assist primer development (Mori and Notomi, 2009). However, primer design programs cannot perform correct primer design under specific circumstances, forcing users to design primers manually (Wong et al., 2018).

Due to the difficulty of primer design and possible cross-reactions of primers, the multiplexing of LAMP is considered less successful than multiplexing PCRs (Dhama et al., 2014).

### 10.2 False positive results

LAMP faces a significant disadvantage in its susceptibility to false positives. Firstly, the increased number of primers can lead to amplification of primer dimers (Wang et al., 2015) or primer secondary structures (Ghosh et al., 2017). However, false positive amplification may be avoided with careful primer design and *in silico* testing (Ghosh et al., 2017). Secondly, the primers may amplify aspecific targets (Aslam et al., 2017). Amplification of secondary primer structures or aspecific products may lead to false positives. As the commonly used sequence-independent (Becherer et al., 2020) or “indirect” (Aslam et al., 2017) evaluation methods cannot differentiate the desired product from nonspecifically amplified products or primer dimer products, it is impossible to identify false positives directly (Liu et al., 2017). This problem can be alleviated by using sequence-specific detection methods (Aslam et al., 2017) that only detect the targeted amplicons. Alternatively, one can rule out false positives through melting curve analysis (Ayukawa et al., 2017).

In addition, thawing reagents on ice, keeping reagents and samples on ice, reducing the length of reaction set-up, and setting up experiments in small batches can help to avoid false positives (Ristaino et al., 2020). The optimal length of incubation is also crucial, as longer incubation results in more false positive results (Ristaino et al., 2020). Sometimes, the presence of betaine in reactions decreases the rate of false positives (Zou et al., 2020).

### 10.3 Cross-contamination

Another serious problem with LAMP is cross-contamination (Niessen and Vogel, 2010). As the method is highly sensitive and as a substantial amount of amplified DNA is produced during the reaction, cross-contamination with only trace amounts of reaction products can lead to false results (Tomita et al., 2008; Takahashi et al., 2014). To avoid cross-contamination, samples and reagents should be handled separately and with care, and reaction mixtures should be set up on distinct clean benches. Tubes should be kept closed after reactions if possible and should be placed in sealable plastic bags (Tomita et al., 2008). Tubes must be opened for agarose gel electrophoresis, or indicators or dyes added after the reaction; in these cases, opening should be done in separate locations (Tomita et al., 2008). Additionally, a technique used in PCR to avoid cross-contamination by using dUTP and uracil DNA glycosylase can also be adapted for use in LAMP (Hsieh et al., 2014).

### 10.4 Amplification inconsistency and problems with colorimetric assays

In some cases, mainly when using *Bsm* polymerase, inconsistent results may be observed (Suleman et al., 2016); this inconsistency may explain why in protocols employing *Bsm* polymerase, a pre-amplification denaturation step is included (e.g., Wang et al., 2019; Zatti et al., 2019), as in the original LAMP protocol (Notomi et al., 2000).

Interpretation of colorimetric LAMP assay results is not always straightforward. For example, color change in HNB is occasionally difficult to distinguish (Tanner et al., 2015) and is also subjective (Lee et al., 2020). The color change of colorimetric mastermixes or indicators may not be as apparent as expected, and intermediate colors can be observed (Omer and Wallenhammar, 2020), complicating the interpretation of results. Regarding HNB, when a slight color change can be observed in the tubes, the reaction can be deemed positive because in such cases agarose gel electrophoresis clearly reveals that DNA amplification has occurred (Palanisamy et al., 2025). By optimizing the reaction temperature and incubation time, color development can be enhanced (Wang et al., 2021).

## 11 Conclusions and outlook

LAMP has become an accepted tool to identify plant pathogenic fungi, and diagnostic primer sets and protocols are available for numerous plant pathogenic fungi. LAMP can often be used after simplified DNA extraction, and it also provides the required specificity and sensitivity, which is almost always superior to PCR.

The available LAMP methods and developments can be grouped into two main functional groups: the instrument-based, precision methods, and the less equipment-demanding, easy-to-use methods. The first group mainly includes instrumental, real-time, fluorescence-based detection assays. These methods are usually more complex or expensive, as equipment for fluorescence detection is required. They may be characterized by increased specificity (**Supplementary Table S1**), and they represent a new opportunity for the assays to become increasingly laboratory-independent. For example, portable, battery-powered fluorescence detectors can be deployed directly in the field (Tomlinson et al., 2013; Si Ammour et al., 2017; Thiessen et al., 2018).

The other set of methods is more cost-efficient, uses cheaper reagents, and requires less elaborate equipment; further, the results are evaluated based on the color change of reaction mixes. SYBR Green or HNB are the most commonly used reagents in those assays. These assays are characterized by easy handling and operation, providing excellent means of quick diagnosis in situations where cost-effectiveness and rapidity are a priority (Niessen et al., 2012), or in less equipped laboratories.

LAMP has its drawbacks and limitations, but these may be overcome with thorough primer design and good laboratory practices. The overwhelming number of publications reporting LAMP assays for the detection of a wide range of pathogens indicates that the advantages of the technology highly outcompete its drawbacks. We expect that numerous new developments in the LAMP method will be available in the near future, and the number of assays will also increase. Eventually, LAMP may reach a level of general popularity in plant pathogenic studies.
