# Supplementary material for: A comprehensive guide to loop-mediated isothermal amplification, an emerging diagnostic tool for plant pathogenic fungi
Source: Front Plant Sci. 2025 May 22;16:1568657. doi: 10.3389/fpls.2025.1568657 (PMC12142221; doi:10.3389/fpls.2025.1568657)
Supplement: Supplementary file 1 [file Table1.pdf]

Supplementary Table 1. List of published LAMP assays developed for the detection of plant pathogenic fungi. SOP stands for several other species.

Reference	Pathogen species	Disease caused	Host plants	Common name of host plants	Target locus	Sensitivity compared to PCR	Analytical sensitivity	Method of results detection
Tomlinson et al. 2007	*Phytophthora ramorum*	sudden oak death, dieback, blight	*Lithocarpus densiflorus, Quercus* spp., SOP	tanoaks, oaks, several other species	rDNA ITS		~10 pg	RT/fluorescent (EvaGreen); PicoGreen
Niessen & Vogel 2010	*Fusarium graminearum*	head blight	*Hordeum vulgare , Triticum aestivum*	barley, common wheat	*gaoA*		1 pg	calcein
Tomlinson et al. 2010a	*Botrytis cinerea*	grey mould	*Rosa* spp., *Pelargonium* spp.	rose, pelargonium, several other species	rDNA IGS		6.5 pg/µl	RT/fluorescent
Tomlinson et al. 2010b	*Phytophthora ramorum*	sudden oak death, dieback, blight	*Lithocarpus densiflorus, Quercus* spp., SOP	tanoaks, oaks, several other species	rDNA ITS			lateral flow device
Tomlinson et al. 2010b	*Phytophthora kernoviae*	dieback, blight	*Rhododendron* spp., *Fagus* spp.	rhododendron, beech	rDNA ITS			lateral flow device
Huang et al. 2011	*Puccinia striiformis* f. sp. *tritici*	stripe (yellow) rust	*Triticum aestivum*	common wheat	β-tubulin		2 pg/µl	SYBR Green
Dai et al. 2012	*Phytophthora sojae*	damping off, root rot	*Glycine max,* other species	soybean, other species	*A3aPro*		10 pg/µl	HNB
Niessen et al. 2012	*Fusarium tricinctum* species complex	head blight		cereals	ATP citrate lyase 1		0.95 pg	calcein
Almasi et al. 2013	*Fusarium oxysporum* f. sp. *lycopersici*	fusarium wilt	*Solanum lycopersicum*	tomato	rDNA 28S	5×	50 ng/µl	several methods
Chen et al. 2013	*Phytophthora melonis*	blight, dieback, rot		several species	*Ypt1*	1000×	10 fg	SYBR Green
Fukuta et al. 2013	*Pythium ephanidermatum*	damping-off	*Solanum lycopersicum*	tomato	rDNA ITS	10×	10 fg	RT/turbidity, turbidity
Jędryczka et al. 2013	*Leptosphaeria maculans*	blackleg or stem canker	*Brassica napus* subsp. *napus*	oilseed rape				RT/fluorescent
Jędryczka et al. 2013	*Leptosphaeria biglobosa*	blackleg or stem canker	*Brassica napus* subsp. *napus*	oilseed rape				RT/fluorescent
Li B et al. 2013	*Fusarium oxysporum* f. sp. *cubense* race 4	fusarium wilt	*Musa* sp.	banana	specific SCAR marker		10 fg	SYBR Green
Peng et al. 2013	*Fusarium oxysporum* f. sp. *niveum*	fusarium wilt	*Citrullus* spp.	watermelon	specific RAPD marker	100×	1.2 pg/µl	RT/fluorescent, SYBR Green
Tao & Cai 2013	*Colletotrichum kahawae*	berry disease	*Coffea arabica*	coffee	Apn2/MAT		80 fg/µl	calcein
Tomlinson et al. 2013	*Guignardia citricarpa*	black spot disease	*Citrus* spp.	lemon	rDNA ITS		60-600 fg	RT/fluorescent
Zhang et al. 2013	*Fusarium oxysporum* f. sp. *cubense*	fusarium wilt	*Musa* sp., *Heliconia* sp.	banana, lobster-claws (toucan beak)	rDNA IGS		0.43 pg/µl	RT/fluorescent, SYBR Green
Duan et al. 2014a	*Sclerotinia sclerotiorum*	white mould	*Brassica napus* subsp. *napus* , SOP	rapeseed, SOP	*Ssos5*	1000×	0.1 fg/µl	HNB
Duan et al. 2014c	*Botrytis cinerea*	grey mould	*Fragaria ananassa, Apium graveolens, Solanum lycopersicum, Cucumus sativus,* SOP	strawberry, celery, tomato, cucumber, SOP	*Bcos5*	10×	1 pg/µl	HNB
Fukuta et al. 2014	*Pythium myriotylum*	root rot		several species	rDNA ITS	equal	100 fg	RT/fluorescent
Moradi et al. 2014	*Verticillium dahliae*	wilt	*Olea europaea,* SOP	olive, several other species	RAPD marker	10×	50 fg, 500 fg	turbidity, HNB, GeneFinder, EtBr, SYBR Premix Ex Taq II dye
Peng et al. 2014	*Fusarium oxysporum* f. sp. *cubense* race 4	wilt	*Musa* sp.	banana	RAPD marker		1000 spores	RT/fluorescent, SYBR Green
Pu et al. 2014	*Fusarium mangiferae*	mango malformation disease	*Mangifera indica*	mango	specific SCAR marker	100×	226 pg/µl	RT/fluorescent, SYBR Green
Takahashi et al. 2014	*Pythium helicoides*	root rot	*Euphorbia pulcherrima* , SOP	poinsettia, several other species	rDNA ITS	equal	100 fg	RT/turbidity, turbidity
Chandra et al. 2015	*Colletotrichum falcatum*	red rot	*Saccharum* spp.	sugarcane	specific SCAR marker	10×	5 ng	SYBR Green
Dong et al. 2015	*Phytophthora capsici*	blight, fruit rot		several species	ITS		100 fg	SYBR Green
Feng et al. 2015	*Pythium irregulare*	seed, stem, root rot, damping off	*Lactuca sativa*	lettuce	rDNA ITS	~equal	100 fg	RT/turbidity
Ghosh et al. 2015	*Fusarium oxysporum* f. sp. *ciceris*	fusarium wilt	*Cicer arietinum*	chickpea	EF-1α	10 000×	10 fg	HNB
Hieno et al. 2015	*Pyrenochaeta lycopersici*	corky root	*Lycopersicon esculentum* and other solanaceous plant species	tomato and other solanaceous plant species	rDNA ITS	equal	10 pg, 100 pg	RT/turbidity
Li B et al. 2015	*Phytophthora nicotianae*	blights, rots	*Nicotiana tabacum, Solanum lycopersicum, Citrus* spp., SOP	tobacco, tomato, lemon, SOP	*Ypt1*	10×	10 fg	calcein
Lu et al. 2015a	*Fusarium oxysporum*	fusarium wilt	*Glycine max*	soybean	*CYP51C*	100×	4 conidia	SYBR Green
Lu et al. 2015b	*Macrophomina phaseolina*	charcoal rot	*Glycine max,* other species	soybean, other species	rDNA ITS		100 pg	SYBR Green
Lu et al. 2015b	*Rhizoctonia solani*	seedling blight	*Glycine max,* other species	soybean, other species	rDNA ITS		10 pg	SYBR Green
Lu et al. 2015c	*Fusarium equiseti*	root rot	*Glycine max*	soybean	CYP51C		10 pg/µl	SYBR Green
Lu et al. 2015c	*Fusarium graminearum*	root rot	*Glycine max*	soybean	CYP51C		100 pg/µl	SYBR Green
Patel et al. 2015	*Rhizoctonia zeae*	leaf and sheath lesion	*Stenotaphrum secundatum, Festuca arundinacea, Agrostis* spp., *Cynodon dactylon, Eremochloa ophiuroides*	St. Augustine grass, tall fescue, bentgrass, Bermuda grass, centipedegrass	rDNA ITS		1 pg	turbidity, SYBR Green, LFD
Patel et al. 2015	*Rhizoctonia solani*	root rot, crown rot, damping off, seed decay, blights, wilts		several species	rDNA ITS		10 fg	turbidity, SYBR Green, LFD
Zhao et al. 2015	*Phytophthora sojae*	damping-off, root rot	*Glycine max,* other species	soybean, other species	*Ypt1*		10 pg	HNB
Ayukawa et al. 2016	*Fusarium oxysporum* f. sp. *lycopersici*	fusarium wilt	*Solanum lycopersicum*	tomato	*SIX4, SIX5*	10×	300 fg	melting curve
Cao et al. 2016	*Pythium inflatum*	stalk rot	*Zea mays*	maize	rDNA ITS		0.1 pg/µl	RT/fluorescent
Chandra et al. 2016	*Puccinia kuehnii*	orange rust	*Saccharum officinarum*	sugarcane	rDNA ITS		100 pg	SYBR Green
Chen et al. 2016	*Ascochyta rabiei*	Ascochyta blight	*Cicer arietinum*	chickpea	ITS	100×	6.01 fg/µl	SYBR Green
Dai et al. 2016	*Phomopsis longicolla*	seed decay, stem blight, stem canker	*Glycine max*	soybean	*TEF1-α*		100 pg/µl	HNB
Gao et al. 2016	*Tilletia indica*	karnal bunt	*Hordeum vulgare*	barley	unique mitochondrial DNA region	equal	10 pg	calcein
Hansen et al. 2016	*Phytophthora infestans*	late blight	*Solanum tuberosum, Solanum lycopersicum*	potato, tomato	Region 86		200 pg	HNB
Kandan et al. 2016	*Colletotrichum capsici*	fruit rot	*Capsicum annuum*	pepper	β-tubulin	10 000×	10 fg/µl	SYBR Green
Katoh et al. 2016	*Colletotrichum gloeosporioides*	anthracnose		several species	rDNA ITS	100×	10 fg	fluorescent reagent, mastermix
Kong et al. 2016	*Plasmopara viticola*	downy mildew	*Vitis vinifera*	grapevine	rDNA ITS	100×	33 fg	HNB
Moghimi et al. 2016	*Alternaria alternata*	Alternaria alternata tangerine pathotype	*Citrus tangerina*	tangerine	*actts2* (enoyl reductase)		<2 pg	SYBR Green
Qiao et al. 2016	*Cylindrocladium scoparium*	dieback	*Eucalyptus* spp.	eucalypts	beta-tubulin	1000×	5 fg	SYBR Green
Shen et al. 2016	*Sporisorium scitamineum*	smut	*Saccharum* spp.	sugarcane	rDNA ITS	100×	2 fg	SYBR Green
Su et al. 2016	*Sporisorium scitamineum*	smut	*Saccharum* spp.	sugarcane	*pep1*	100×	10^4 copies	SYBR Green
Thiessen et al. 2016	*Erysiphe necator*	powdery mildew	*Vitis vinifera*	grapevine	rDNA ITS2		1-10 conidia	turbidity
Yao et al. 2016	*Didymella bryoniae*	gummy stem blight	Cucurbitaceae	cucurbits	specific SCAR marker	1000×	0.1 fg	calcein
Zhang et al. 2016	*CColletotrichum fragariae*	anthracnose fruit rot	*Fragaria ananassa*	strawberry	rDNA ITS		20 pg	PicoGreen
Zhang et al. 2016	*Colletotrichum gloeosporioides*	anthracnose fruit rot	*Fragaria ananassa*	strawberry	rDNA ITS		20 pg	PicoGreen
Zhang et al. 2016	*Colletotrichum acutatum*	anthracnose fruit rot	*Fragaria ananassa, Citrus* spp., SOP	strawberry, citrus, SOP	β-tubulin 2		200 pg	PicoGreen
Zhang et al. 2016	*Colletotrichum acutatum*	anthracnose fruit rot	*Fragaria ananassa, Citrus* spp., SOP	strawberry, citrus, SOP	rDNA ITS		20 pg	PicoGreen
Aggarwal et al. 2017	*Puccinia striiformis* f. sp. *tritici*	stripe (yellow) rust	*Triticum aestivum*	common wheat	Ketopantoate reductase	10×	1 pg	HNB
Aslani et al. 2017	*Verticillium dahliae*	wilt	*Olea europaea*	olive tree				EtBr
Cao et al. 2017	*Ustilago maydis*	smut	*Zea mays*	corn	Pep1	200×	44 fg/µl	RT/fluorescent, SYBR Green
Chen et al. 2017	*Colletotrichum gloeosporioides*	anthracnose	*Anoectochilus roxburghii*	marbled jewel orchid	rDNA ITS	1000×	10 fg	calcein
Fukuta et al. 2017	*Fomitiporia torreyae*	leaf malformation, leaf dwarfism	*Pyrus pyrifolia* var. *culta*	pear	rDNA ITS		100 fg	turbidity, precipitate fluorescence
Fukuta et al. 2017	*Fulviformes umbrinellus*	leaf malformation, leaf dwarfism	*Pyrus pyrifolia* var. *culta*	pear	rDNA ITS		100 fg	turbidity, precipitate fluorescence
Ghosh et al. 2017	*Rhizoctonia bataticola*	dry root rot	*Cicer arietinum*	chickpea	rDNA ITS	100×	10 fg	SYBR Green
Khan et al. 2017	*Phytophthora infestans*	late blight	*Solanum tuberosum, Solanum lycopersicum*	potato, tomato	Ypt1	1000×	128 fg	calcein
Rocha et al. 2017	*Macrophomina phaseolina*	charcoal rot	*Phaseolus vulgaris,* SOP	common bean, SOP	specific SCAR marker		1 pg	HNB
Shen et al. 2017	*Pythium ultimum*	root rot, damping-off	*Triticum aestivum, Glycine max, Cucumis sativus, Nicotiana tabacum,* SOP	wheat, soybean, cucumber, tobacco, SOP	unique spore wall protein gene	1000×	1 pg	HNB
Si Ammour et al. 2017	*Phytophthora infestans*	late blight	*Solanum* spp.	potato, solanaceous plants	rDNA ITS		50 fg/µl	RT/fluorescent
Tian et al. 2017a	*Colletotrichum truncatum*	anthracnose	*Glycine max*	soybean	RPB1		100 pg/µl	SYBR Green
Tian et al. 2017b	*Didymella bryoniae*	gummy stem blight	Cucurbitaceae	cucurbits	*RPB2*		10 pg	calcein
Tomlinson & Boonham 2015, Harrison et al. 2017	*Hymenoscyphus fraxineus* (syn. *Chalara fraxinea*)	ash dieback	*Fraxinus excelsior*	common ash			7 pg	RT/fluorescent
Villari et al. 2017	*Magnaporthe oryzae*	gray leaf spot	*Lolium perenne*	ryegrass	specific marker			RT/fluorescent
Wang et al. 2017	*Colletotrichum gloeosporioides*	anthracnose		cereals, legumes, vegetables, perennial crops, tree fruits	glutamine synthetase		1 pg	SYBR Green
Xu et al. 2017	*Fusarium asiaticum*	head blight	*Triticum* sp., other Poaceae species	wheat and other cereals	CYP51C		100 pg/µl	HNB
Zeng et al. 2017	*Fusarium culmorum*	head blight, ear rot, crown rot	*Glycine max* , Poaceae, SOP	soybean, cereals and several other species	*CYP51C*		100 pg	HNB
Akul et al. 2018	*Ganoderma boninense*	basal stem rot	*Elaeis guineensis*	oil palm	MnSOD			agarose gel
Karakkat et al. 2018a (method) and 2018b (primers)	*Gaeummanomyces avenae*	take-all patch	*Agrostis stolonifera, Festuca* spp., *Poa* spp.	creeping bentgrass, fescues, meadow-grasses	18S rDNA		1 pg	colorimetric mastermix
Karakkat et al. 2018a (method) and 2018b (primers)	*Ophiosphaerella korrae*	necrotic ring spot	*Poa pratensis, P. annua, Festuca* spp.	common meadow-grass, annual meadow-grass, fescues	18S rDNA		1 fg	colorimetric mastermix
Karakkat et al. 2018a (method) and 2018b (primers)	*Magnaporthiopsis poae*	summer patch	*Poa pratensis, P. annua, Festuca* spp., *Agrostis stolonifera*	common meadow-grass, annual meadow-grass, fescues, creeping bentgrass	18S rDNA		100 fg	colorimetric mastermix
Khan et al. 2018	*Alternaria solani*	early blight	*Solanum tuberosum, Solanum lycopersicum*	potato, tomato	histidine kinase HK1	10×	10 pg	SYBR Green
King et al. 2018	*Pyrenopeziza brassicae*	light leaf spot	*Brassica* spp.	brassicas	ITS		1 pg	RT/fluorescent
King et al. 2018	*Pyrenopeziza brassicae*	light leaf spot	*Brassica* spp.	brassicas	β-tubulin		10 pg	RT/fluorescent
Lan et al. 2018	*Fusarium oxysporum* f. sp. *cucumerinum*	fusarium wilt	*Cucumis sativus*	cucumber	specific RAPD marker	1000×	100 fg	SYBR Green
Madihah et al. 2018	*Ganoderma boninense, G. zonatum, G. miniatocinctum*	basal stem rot	*Elaeis guineensis*	oil palm	*BUG1*	10×	2 pg	RT/fluorescent
Malapi-Wight et al. 2018	*Calonectria henricotiae, C. pseudonaviculata*	boxwood blight	*Buxus* spp.	boxwood	unique non-coding sequence		100 pg	capillary gel electrophoresis
Malapi-Wight et al. 2018	*Calonectria henricotiae, C. pseudonaviculata*	boxwood blight	*Buxus* spp.	boxwood	patatin-like phospholipase		100 pg	capillary gel electrophoresis
Manjunatha et al. 2018	*Puccinia triticina*	leaf rust	*Triticum aestivum*	wheat	specific SCAR marker	500×	100 fg	HNB, EtBr
Ortega et al. 2018a	*Fusarium oxysporum* f. sp. *lactucae*	fusarium wilt	*Lactuca sativa*	lettuce	specific SCAR marker		≤999 pg	RT/fluorescent
Ortega et al. 2018b	*Fusarium fujikuroi*	bakanae disease	*Oryza sativa*	rice	elongation factor 1-α		100-999 fg	RT/fluorescent
Ortega et al. 2018b	*Magnaporthe oryzae*	rice blast	*Oryza sativa*	rice	calmodulin		10-99 pg	RT/fluorescent
Pieczul et al. 2018	*Tilletia caries*	common bunt	*Triticum aestivum*	wheat	rDNA IGS (IGS 2)		1 pg	RT/fluorescent, Evagreen
Pieczul et al. 2018	*Tilletia controversa*	dwarf bunt	*Triticum aestivum*	wheat	rDNA IGS (IGS 2)		1 pg	RT/fluorescent, Evagreen
Pieczul et al. 2018	*Tilletia laevis*	common bunt	*Triticum aestivum*	wheat	rDNA IGS (IGS 2)		1 pg	RT/fluorescent, Evagreen
Rong et al. 2018	*Fusarium proliferatum*	bakanae disease	*Oryza sativa*	rice	RED1		1 ng	HNB
Rong et al. 2018	*Fusarium fujikuroi*	bakanae disease	*Oryza sativa*	rice	rDNA IGS		100 pg	HNB
Sillo et al. 2018	*Heterobasidion irregulare*	root rot	*Pinus sylvestris* and other conifers	pine trees and other conifers	unique sequence (Cytochrome P450 monooxygenase)		~20 pg	RT/fluorescent
Thangavelu & Devi 2018	*Pseudocercospora eumusae*	leaf spot	*Musa* sp.	banana	specific SCAR marker	100×	10 pg	SYBR Green
Thiessen et al. 2018	*Erysiphe necator*	powdery mildew	*Vitis vinifera*	grapevine	rDNA ITS2		1 conidium	RT/fluorescent
Yang et al. 2018	*Ustilaginoidea virens*	false smut (green smut)	*Oryza sativa*	rice	UvG-β1	1000×	1 pg	HNB, SYBR Green
Yasuhara-Bell et al. 2018	*Magnaporthe oryzae*	blast	Poaceae	grasses	PoT2 transposon		5 pg	RT/fluorescent
Yasuhara-Bell et al. 2018	*Magnaporthe oryzae* pathotype *Triticum*	wheat blast	*Triticum aestivum*	wheat	MoT3 retinol dehydrogenase		5 pg	RT/fluorescent
Aglietti et al. 2019	*Ceratocystis platani*	canker stain disease	*Platanus* sp.	plane trees	rDNA ITS		0.02 pg/µl	RT/fluorescent
Aglietti et al. 2019	*Phytophthora ramorum*	sudden oak death	*Quercus* sp., *Larix kaempferi*	oaks, Japanese larch	rDNA ITS		0.128 pg/µl	RT/fluorescent
Almasi 2019	*Fusarium oxysporum* f. sp. *melonis*	fusarium wilt	*Citrullus* spp.	watermelon	elongation factor 1 alpha	100×		agarose gel, HNB
Dai et al. 2019	*Phytophthora cinnamomi*	root rot, crown rot, dieback	*Cedrus deodara, SOP*	deodar cedar, SOP	unique gene *Pcinn100006*	10×	0.1 ng	turbidity, HNB
Feng H et al. 2019	*Pythium spinosum*	seed, seedling and root rot	*Glycine max, Arachis hypogaea, Triticum aestivum*	soybean, peanut, wheat	ITS (ITS2)		100 pg	HNB
Feng W et al. 2019a	*Phytophthora speudolactucae*	wilt	*Lactuca sativa*	lettuce	*cox1*	~equal	100 fg	RT/turbidity
Feng W et al. 2019a	*Pythium spinosum*	damping off	*Lactuca sativa*	lettuce	rDNA ITS	~equal	10 fg	RT/turbidity
Feng W et al. 2019a	*Pythium uncinulatum*	damping off	*Lactuca sativa*	lettuce	rDNA ITS	~equal	100 fg	RT/turbidity
Feng W et al. 2019b	*Phytophthora colocasiae*	leaf blight	*Colocasia esculenta*	taro	*Ypt1*		100 fg	RT/turbidity, SYBR Green
Kaczmarek et al. 2019	*Uromyces betae*	rust	*Beta vulgaris*	sugar beet	cytochrome b		10 pg	RT/fluorescent
Lau et al. 2019	*Pyricularia oryzae*	rice blast	*Oryza sativa*	rice	Pot2 unique transposon		0.5 pg	agarose gel
Li GR et al. 2019	*Phytophthora cambivora*	wilt, root, stem and fruit rot	several species		*Ypt1*	10000×	20 ag	turbidity, SYBR Green
Li GR et al. 2019	*Phytophthora syringae*	wilt, root, stem and fruit rot	several species		rDNA ITS	10000×	20 ag	turbidity, SYBR Green
Li GR et al. 2019	*Phytophthora hibernalis*	wilt, root, stem and fruit rot	several species		*Enol*	10000×	20 ag	turbidity, SYBR Green
Li L et al. 2019	*Magnaporthe oryzae*	rice blast	*Oryza sativa*	rice	MGG_04322		10 pg	HNB, RT detection
Ordóñez et al. 2019	*Fusarium odoratissimum (Fusarium oxysporum* f. sp. *cubense* tropical race 4)	wilt	*Musa* sp.	banana species	unique sequence (*SeqA*)		1 pg	RT/fluorescent
Ortega et al. 2019	*Monilinia fructicola*	blight, brown rot	*Prunus, Malus* and *Pyrus* species	plums, cherries, and allies, apple and pear trees	cytochrome b		≤ 999 fg	RT/fluorescent
Ortega et al. 2019	*Monilinia laxa*	blight, brown rot	*Prunus, Malus* and *Pyrus* species	plums, cherries, and allies, apple and pear trees	specific SCAR marker		≤ 99 fg	RT/fluorescent
Parkinson et al. 2019	*Calonectria ilicicola*	black root rot	*Persea americana*	avocado	beta-tubulin		1 pg	RT/fluorescent
Parkinson et al. 2019	*Dactylonectria macrodidyma*	black root rot	*Persea americana*	avocado	histone H3		0.01 ng	RT/fluorescent
Parkinson et al. 2019	*Dactylonectria sp.*	black root rot	*Persea americana*	avocado	histone H3		0.1 ng	RT/fluorescent
Shan et al. 2019	*Fusarium temperatum*	ear and stalk rot	*Zea mays*	maize	28S rDNA		10 pg	SYBR Green
Sunani et al. 2019	*Fusarium fujikuroi*	bakanae disease	*Oryza sativa*	rice	NRPS31		10 pg/µl	HNB
Verma et al. 2019	*Phytophthora infestans*	late blight	*Solanum tuberosum, Solanum lycopersicum*	potato, tomato	rDNA ITS	100×	1 pg/µl	SYBR Green
Wu et al. 2019	*Colletotrichum gloeosporioides*	anthracnose	*Fragaria ananassa,* SOP	stawberry/several species	beta-tubulin	10×	0.1 pg; 1 pg	agarose gel, HNB
Yan et al. 2019	*Ustilago tritici*	loose smut	*Triticum* sp.	wheat	rDNA ITS, rDNA LSU		100 fg	SYBR Green
Yang et al. 2019	*Alternaria alternata*	black spot disease	*Pyrus* sp.	pear	*cytb*	1000×	1 pg	SYBR Green
Zhang et al. 2019	*Fusarium fujikuroi*	bakanae disease	*Oryza sativa*	rice	NRPS31	100×	10 fg	HNB
Chen et al. 2020	*Colletotrichum gloeosporioides*	anthracnose	*Canarium album*	Chinese olive	translation elongation factor alpha 1		0.4 fg	SYBR Green
Choudhary et al. 2020	*Rhizoctonia solani* AG-1 IA	sheath blight	*Oryza sativa, Zea mays,* Poaceae, SOP	rice, maize, gramineous plants, other hosts	polygalacturonase		1.65 fg/µl	colorimetric mastermix
Gupta et al. 2020	*Fusarium graminearum*	head blight	*Triticum aestivum*	wheat	unique sequence	equal	100 fg	HNB, EtBr
Hamilton et al. 2020	*Raffaelea lauricola*	laurel vascular wilt	Lauraceae	lauraceous hosts	β-tubulin		0.5 pg	RT/fluorescent
Kong et al. 2020	*Phytophthora infestans*	late blight	*Solanum tuberosum*	potato	specific multiple copy DNA sequences		1 pg	HNB
Lan et al. 2020	*Colletotrichum gloeosporioides*	anthracnose	*Psidium guajava*	guava	β-tubulin 2	~1000×	10 fg	SYBR Green
Lee et al. 2020	*Rosellinia necatrix*	white root rot	*Pyrus* sp.	pear, various other hosts	unique sequence		10 fg	RT/fluorescent
Marimuthu et al. 2020	*Plasmopara viticola*	downy mildew	*Vitis vinifera*	grapevine	*CesA4*		20 fg	HNB
Omer & Wallenhammar 2020	*Leptosphaeria biglobosa*	blackleg	*Brassica napus* subsp. *napus*	winter oil seed rape	polyketide synthase *PKS21*		100 fg	RT/fluorescent, colorimetric mastermix
Omer & Wallenhammar 2020	*Leptosphaeria maculans*	blackleg	*Brassica napus* subsp. *napus*	winter oil seed rape	non-ribosomal peptide synthase gene *sirP*		180 fg	RT/fluorescent, colorimetric mastermix
Omer & Wallenhammar 2020	*Leptosphaeria maculans*	blackleg	*Brassica napus* subsp. *napus*	winter oil seed rape	polyketide synthase *PKS5*		30 pg	RT/fluorescent, colorimetric mastermix
Ristaino et al. 2020	*Phytophthora infestans*	late blight	*Solanum tuberosum, Solanum lycopersicum*	potato, tomato	rDNA ITS	10×	1 pg/µl	HNB, Eva Green, LFD
Shrestha et al. 2020	*Cercospora beticola*	leaf spot	*Beta vulgaris*	sugar beet	rDNA ITS		100 fg	colorimetric mastermix
Stehlíková et al. 2020	*Fusarium circinatum*	pitch canker	Pinaceae	conifers	EF1α		0.5 pg	RT/fluorescent with mastermix and probe
Wang et al. 2020	*Fusarium proliferatum*	ear and kernel rot	*Zea mays* , SOP	maize/ several additional species	TEF-1α		10 pg	SYBR Green
Winkworth et al. 2020	*Phytophthora agathidicida*	root rot, bleeding cankers	*Agathis australis*	kauri	apocytochrome b		1 fg	RT/fluorescent
Yang et al. 2020	*Coniella granati*	twig blight, crown rot	*Punica granatum*	pomegranate	EF1α		1 pg	SYBR Green
Zou et al. 2020	*Fusarium oxysporum* f. sp. *conglutinans*	fusarium wilt	Brassicaceae	cruciferous crops	endopolygalacturonase		100 pg	RT/fluorescent
Feng et al. 2021	*Pythium terrestris*	damping-off, root rot	*Glycine max*	soybean	*M90* Puf family RNA-binding protein gene		100 pg/ul	SYBR Green
Feng et al. 2021	*Candidatus Pythium huanghuaiense*	seed and root rot	*Glycine max*	soybean	*M90* Puf family RNA-binding protein gene		100 pg/ul	SYBR Green
Feng et al. 2021	*Pythium spinosum*	wilt, dieback, damping-off, root rot	*Glycine max* , SOP	soybean, other plants	*M90* Puf family RNA-binding protein gene		100 pg/ul	SYBR Green
Katoh et al. 2021	*Fusarium oxysporum f. sp. fragariae*	vascular wilt	*Fragaria ananassa*	strawberry	unique sequence		100 pg	colorimetric mastermix (containing calcein)
King et al. 2021	*Oculimacula yallundae*	eyespot	*Triticum aestivum, Hordeum vulgare, Avena sativa*	wheat, barley, rye	β-tubulin, MAT1-1, MAT1-2		1 pg	colorimetric mastermix
King et al. 2021	*Oculimacula acuformis*	eyespot	*Triticum aestivum, Hordeum vulgare, Avena sativa*	wheat, barley, rye	β-tubulin, MAT1-1, MAT1-2		1 pg	colorimetric mastermix
Kong et al. 2021	*Peronophythora litchii*	downy blight	*Litchi chinensis*	lychee	M90	100×	10 pg	SYBR Green
Liu Y et al. 2021	*Colletotrichum* spp.	anthracnose	*Fragaria ananassa* , SOP	strawberry/several hosts	β-tubulin 2		100 pg	HNB
Liu Z et al. 2021	*Cercospora canescens*	leaf spot	*Vigna radiata*	mungbean	cytb		100 pg	SYBR Green, EvaGreen fluorescence
Myrholm et al. 2021	*Dothistroma septosporum*	needle blight	*Pinus* sp.	pine trees	β-tubulin 2		1 pg	RT/fluorescent
Prasannakumar et al. 2021	*Magnaporthe oryzae*	blast	*Oryza sativa*	rice	RNA polymerase II largest subunit	10000×	100 fg	EtBr, basic fuchsin
Prasannakumar et al. 2021	*Sarocladium oryzae*	sheat rot	*Oryza sativa*	rice	RNA polymerase II largest subunit	10000×	100 fg	EtBr, basic fuchsin
Ren et al. 2021	*Marssonina coronaria*	blotch	*Malus domestica*	apple	ITS		100 fg	HNB
Sanna et al. 2021	*Fusarium fujikuroi*	bakanae disease	*Oryza sativa*	rice	EF1α		100 fg	RT/fluorescent
Sedaghatjoo et al. 2021	*Tilletia controversa*	dwarf bunt	*Triticum aestivum*	wheat	species-specific genome region		5 pg	neutral red

Siegieda et al. 2021	*Phytophthora cactorum*	crown rot, leather rot	*Fragaria ananassa* , other berry fruits	strawberry, other berry fruits	EF1α		300 fg	RT/fluorescent, SYBR Green
Siegieda et al. 2021	*Phytophthora* sp.	crown rot, leather rot	*Fragaria ananassa* , other berry fruits	strawberry, other berry fruits	EF1α		0.3 ng	RT/fluorescent, SYBR Green
Tong et al. 2021	*Phytophthora cinnamomi*	dieback, basal stem necrosis, stem canker	*Carya cathayensis*	chinese hickory	unique gene *Pcinn100006* (Dai *et al.* 2019)		80 pg	HNB
Wang et al. 2021	*Phytopythium vexans*	seedling damping-off, brown root rot, crown rot	*Ginkgo biloba, Citrus* spp., *Prunus* spp., *Vitis vinifera, Acer rubrum* , SOP	gingko, lemon, kiwifruit, cherry, grapevine, red maple, SOP	rDNA ITS	100×	1 pg	HNB
Wang et al. 2021	*Ustilaginoidea virens*	false smut (green smut)	*Oryza sativa*	rice	*APN1* aminopeptidase	10×	100 pg	HNB
Xiao & Li 2021	*Fusarium oxysporum*	soft rot	*Dendrobium officinale*	dendrobium	EF1α		5 fg	SYBR Green
Xiong et al. 2021	*Marssonina brunnea*	black spot disease	*Populus* spp.	poplars	rDNA ITS	100×	10 pg	HNB
Xu L et al. 2021	*Valsa mali*	Valsa canker	*Malus domestica*	apple	elongation factor 1α		1 ng	SYBR Green, EvaGreen fluorescence
Zhou et al. 2021	*Venturia carpophila*	scab	*Prunus persica*	peach	rDNA ITS	100×	56.6 fg	colorimetric mastermix
Choudhary et al. 2022	*Sarocladium oryzae*	sheath rot	*Oryza sativa*	rice	actin		1.6 fg/µl	colorimetric mastermix
Hu et al. 2022	*Pyrenophora graminea*	leaf stripe	*Hordeum vulgare*	barley	pig 14	10×	10 pg/µl	SYBR Green
Lakshmi et al. 2022	*Bipolaris oryzae*	brown spot	*Oryza sativa*	rice	glycoside hydrolase family 13 protein		100 fg	HNB
Lan et al. 2022	*Mycocentrospora acerina*	round leaf spot	*Panax notoginseng*	notoginseng	rDNA ITS		10 fg	SYBR Green
Liu et al. 2022	*Alternaria alternata*	blotch	*Malus domestica*	apple	*aapg-1* endopolygalacturonase	equal	1 fg	SYBR Green
Logeshwari et al. 2022	*Sarocladium oryzae*	sheath rot	*Oryza sativa*	rice	β-tubulin		10 fg	HNB
Rizzo et al. 2022	*Geosmithia morbida*	thousand cankers disease	*Juglans nigra*	walnut	kinesin		3.2 pg/µl	HNB, RT/fluorescent
Sadallah et al. 2022	*Pleurostoma richardsiae*	dieback, cankers, wilting	*Olea europaea, Vitis vinifera*	olive tree, grapevine	rDNA IGS		75 pg/µl	RT/fluorescent
Sun et al. 2022	*Phoma macdonaldii*	black stem	*Helianthus annuus*	sunflower	rDNA ITS		100 fg	colorimetric mastermix
Tonka et al. 2022	*Armillaria ostoyae*	decline	*Picea abies*	Norway spruce	*TEF-1α*		1 pg	RT/fluorescent with mastermix and probe
Wang et al. 2022	*Fusarium acuminatum*	root rot	*Astragalus membranaceus*	Mongolian milkvetch	*TEF-1α*		100 fg/µl	SYBR Green
Wang et al. 2022	*Fusarium solani*	root rot	*Astragalus membranaceus*	Mongolian milkvetch	*TEF-1α*		1 pg/µl	SYBR Green
Yang L et al. 2022	*Phomopsis amygdali*	shoot blight	*Prunus persica*	peach	*GME6801* (species-specific gene)	100×	50 pg	SYBR Green
Yang X et al. 2022	*Plasmodiophora brassicae*	clubroot	Brassicaceae	cruciferous crops	partial rDNA 18S-ITS1	100×	1 fg	colorimetric mastermix
Zhang Han et al. 2022	*Arthrinium phaeospermum*	wilting, blight	*Bambusa pervariabilis* x *Dendrocalamopsis grandis*	(a cultivated bamboo hybrid)	*APZ1300015*	10×	10 pg/µl	HNB
Zhang Hao et al. 2022	*Phellinus noxius*	brown root rot	*Acacia confusa* , *Ficus microcarpa* , *Prunus persica* , urban trees	acacia petit feuille, Indian laurel, pear, urban trees				
Achari et al. 2023	*Fusarium oxysporum* f. sp. *ciceris*	fusarium wilt	*Cicer arietinum*	chickpea	forma specialis-specific genomic region		9 pg/µl	RT/fluorescent
Ghimire et al. 2023	*Phytopythium vexans*	seedling damping-off, brown root rot, crown rot	*Ginkgo biloba, Citrus* spp., *Prunus* spp., *Vitis vinifera, Acer rubrum* , SOP	gingko, lemon, kiwifruit, cherry, grapevine, red maple, SOP	rDNA LSU		102 fg	RT/fluorescent, colorimetric mastermix
Hong-min et al. 2023	*Heterobasidion annosum*	root rot, butt rot	*Pinus* sp.	pine trees	GAPDH		100 pg/µl	HNB
Huang et al. 2023	*Clarireedia* spp.	dollar spot	Poaceae	grasses	beta-tubulin	100×	2×10^4 copies/µl	HNB
Ouyang et al. 2023	*Phakopsora pachyrhizi*	rust	*Glycine max*	soybean	*Phapa_6409908* (species-specific gene)		10 pg	SYBR Green
Vielba-Fernández et al. 2023	*Botrytis fragariae*	gray mold	*Fragaria ananassa*	strawberry	*NEP2* (species-specific gene)	equal	210 pg/µl	RT/fluorescent
Zhang J et al. 2023	*Globisporangium sylvaticum*	root rot, wilt, blight, tuber rot	*Zea mays, Lactuca sativa, S. tuberosum* , SOP	maize, lettuce, potato, other hosts	rDNA ITS	10×	1 pg	RT/fluorescent, SYBR Green
Zhang Y et al. 2023	*Ustilaginoidea virens*	false smut (green smut)	*Oryza sativa*	rice	ustiloxins biosynthetic gene (species-specific gene)	25×	6.4 spores/mL	RT/fluorescent
Marek et al. 2024	*Phaeoacremonium minimum*	esca	*Vitis vinifera*	grapevine	putative 14-alpha sterol demethylase protein (species-specific gene)	less sensitive	100 pg	RT/fluorescent, neutral red
Marek et al. 2024	*Phaeomoniella chlamydospora*	esca	*Vitis vinifera*	grapevine	putative carboxypeptidase s1 (species-specific gene)	1.6×	1 pg	RT/fluorescent, neutral red
Marek et al. 2024	*Fomitiporia mediterranea*	esca	*Vitis vinifera*	grapevine	WD40 repeat-like protein gene (species-specific gene)	less sensitive	100 pg	RT/fluorescent, neutral red
Zou et al. 2024	*Colletotrichum siamense*	anthracnose	*Chamelia chinensis*	tea	calmodulin	10×	1 pg	RT/fluorescent, SYBR Green
Dai et al. 2024	*Aspergillus niger*	crown rot, root rot	*Arachis hypogaea*	peanut	GOD		5.1×10^{-7} ng/µL plasmid DNA	RT/fluorescent, SYBR Green
Tu et al. 2024	*Didymella segeticola*	tea leaf spot	*Camellia sinensis*	tea plant	zinc finger protein (species-specific sequence)		1 fg/µL	SYBR Green
Yeni et al. 2024	*Plasmopara halstedii*	downy mildew	*Helianthus annuus*	sunflower	rDNA LSU		0.5 pg/µl	HNB, SYBR Green, neutral red, thiazol green
Zhang et al. 2024	*Phytophthora infestans*	late blight	*Solanum tuberosum*	potato	extracellular protease inhibitor 12 (*Epi12*)		10 pg	SYBR Green
Zhang et al. 2024	*Alternaria solani*	early blight	*Solanum tuberosum*	potato	β-tubulin		100 fg	SYBR Green
Zhang et al. 2024	*Fusarium graminearum*	dry rot	*Solanum tuberosum*	potato	TEF 1α		1 pg	SYBR Green
Zhang et al. 2024	*Rhizoctonia solani*	black spot	*Solanum tuberosum*	potato	rDNA ITS		10 pg	SYBR Green
Palanisamy et al. 2025.	*Podosphaera xanthii*	powdery mildew	Cucurbitaceae	cucurbits	rDNA ITS		15 fg	HNB
Palanisamy et al. 2025.	*Pseudoperonospora cubensis*	downy mildew	Cucurbitaceae	cucurbits	*CesA2*		150 fg	HNB
