# Supplementary material for: A comprehensive guide to loop-mediated isothermal amplification, an emerging diagnostic tool for plant pathogenic fungi
Source: Front Plant Sci. 2025 May 22;16:1568657. doi: 10.3389/fpls.2025.1568657 (PMC12142221; doi:10.3389/fpls.2025.1568657)
Supplement: Supplementary file 2 [file Table2.pdf]

Supplementary Table 2. List of published SNP-specific LAMP assays developed for the detection of certain SNPs in plant pathogenic fungi. SOP stands for several other species.

Reference	*Pathogen species*	*Disease caused*	*Host plants*	*Common name of host plants*	*Target locus*	*SNP*	*SNP associated with*	*Sensitivity compared to PCR*	*Analytical sensitivity*	*Method of results detection*
Duan et al. 2014b	*Fusarium graminearum*	head blight	*Hordeum vulgare, Triticum aestivum*	barley, wheat	*β_2-tubulin*	F167Y	carbendazim resistance			HNB
Duan et al. 2015	*Sclerotinia sclerotiorum*	white mould	*Brassica napus* subsp. *napus*, SOP	rapeseed, SOP	*β-tubulin*	E198A	benzimidazole resistance	1000x		HNB
Duan et al. 2016a	*Fusarium asiaticum*	head blight	*Hordeum vulgare, Triticum aestivum*	barley, wheat and other cereals	*β_2-tubulin*	F200Y	benzimidazole resistance	100x		HNB
Duan et al. 2016b	*Sclerotinia sclerotiorum*	white mould	*Brassica napus* subsp. *napus*, SOP	rapeseed, SOP	*β-tubulin*	F200Y	carbendazim resistance	10000x		HNB
Ayukawa et al. 2017	*Fusarium oxysporum* f. sp. *lycopersici*	wilt	*Solanum lycopersicum,* SOP	tomato, SOP	*SIX3*	G121A, T122A, G134A, C146T	distinct pathogen races	10x		probes/melting curve
Hu et al. 2017	*Botrytis cinerea*	grey mould	*Fragaria ananassa,* SOP	strawberry, SOP	*cytochrome b*	G143A	quinone outside inhibitor resistance		10 pg	HNB
Duan et al. 2018a	*Botrytis cinerea*	grey mould	*Fragaria ananassa, Solanum lycopersicum, Cucumis sativus,* SOP	strawberry, tomato, cucumber, SOP	*β-tubulin*	F200Y	benzimidazole resistance	100x		HNB
Duan et al. 2018b	*Botrytis cinerea*	grey mould		several species	*β-tubulin*	E198A, E198K, E198V	benzimidazole resistance	10x		HNB
Fan et al. 2018	*Botrytis cinerea*	grey mould		several species	*sdhB*	H272R	succinate dehydrogenase inhibitor resistance			SYBR Green I
Komura et al. 2018	*Fusarium graminearum*	head blight	*Hordeum vulgare, Triticum aestivum*	barley, wheat	*β_2-tubulin*	F167Y, E198Q, F200Y	methyl benzimidazole carbamate resistance			FRET probe/melting curve
Fan et al. 2019	*Botrytis cinerea*	grey mould		several species	*β-tubulin*	E198A, E198K	benzimidazole resistance			SYBR Green I
Fan et al. 2019	*Botrytis cinerea*	grey mould		several species	*β-tubulin*	E198V	benzimidazole resistance	100x		SYBR Green I
Liu et al. 2019	*Botrytis cinerea*	grey mould	*Fragaria ananassa,* SOP	strawberry, SOP	*β-tubulin*	E198A	benzimidazole resistance		1 ng	HNB
Vielba-Fernández et al. 2019	*Podosphaera xanthii*	powdery mildew	*Cucumis sativus*, SOP	cucumber, SOP	*β-tubulin*	E198A	methyl benzimidazole carbamate resistance	100x		HNB
Wu et al. 2019	*Colletotrichum gloeosporioides*	anthracnose	*Fragaria ananassa,* SOP	strawberry, SOP	*cytochrome b*	G143A	quinone outside inhibitor resistance	10x	10 pg	HNB
Shrestha et al. 2020	*Cercospora beticola*	leaf spot	*Beta vulgaris*	sugar beet	*cytochrome b*	G143A	quinone outside inhibitor resistance			colorimetric mastermix
Zhu et al. 2020	*Corynespora cassiicola*	leaf spot, target spot		several species	*sdhC*	N75S	succinate dehydrogenase inhibitor resistance	~equal	8.8 fg	SYBR Green I
Vielba-Fernández et al. 2021	*Podosphaera xanthii*	powdery mildew	*Cucumis sativus*, SOP	cucumber, SOP	*sdhC*	A86V, G151R	fluopyram and boscalid resistance			mastermix with fluorescent dye
Shen et al. 2022	*Didymella bryoniae*	gummy stem blight	*Citrullus* spp.	watermelon	*β-tubulin*	E198A	methyl benzimidazole carbamate resistance		1 ng/µl	HNB
Poti et al. 2023	*Colletotrichum truncatum*	anthracnose	*Glycine max*	soybean	*cytochrome b*	G143A	quinone outside inhibitor resistance		1 ng	HNB
